# Cannabidiol attenuates pulmonary arterial hypertension by improving vascular smooth muscle cells mitochondrial function

**DOI:** 10.7150/thno.55571

**Published:** 2021-03-11

**Authors:** Xiaohui Lu, Jingyuan Zhang, Huijiao Liu, Wenqiang Ma, Leo Yu, Xin Tan, Shubin Wang, Fazheng Ren, Xiru Li, Xiangdong Li

**Affiliations:** 1 State Key Laboratory of Agrobiotechnology, College of Biological Sciences, China Agricultural University, Beijing 100193, China.; 2 Yunnan Hempmon Pharmaceuticals Co. Ltd., Beijing, 100010, China.; 3 Hanma Investment Group Co., Ltd., Beijing, 100010, China.; 4 Department of Nutrition and Health, China Agricultural University, Beijing 100193, China.; 5 Department of Surgery, Chinese PLA General Hospital, Beijing, 100071, China.; 6 Department of Reproduction and Gynecological Endocrinology, Medical University of Bialystok, Bialystok, Poland.

**Keywords:** Cannabinoids, Pulmonary arterial hypertension, Antioxidant, Mitochondria networks, Glycolysis.

## Abstract

**Rationale:** Pulmonary arterial hypertension (PAH) is a chronic disease associated with enhanced proliferation of pulmonary artery smooth muscle cells (PASMCs) and dysfunctional mitochondria, and the clinical therapeutic option for PAH is very limited. Recent studies showed that cannabidiol (CBD), a non-psychoactive constituent of cannabinoids, possessed antioxidant effect towards several cardiovascular diseases, whereas the mechanistic effect of CBD in PAH is unknown.

**Methods:** In this study, the effects of CBD in PAH were determined by analyzing its preventive and therapeutic actions in PAH rodent models in vivo and PASMCs' proliferation test in vitro. Additionally, CBD's roles in mitochondrial function and oxidant stress were also assessed in PASMCs.

**Results:** We found that CBD reversed the pathological changes observed in both Sugen-hypoxia and monocrotaline-induced PAH rodent models in a cannabinoid receptors-independent manner. Our results also demonstrated that CBD significantly inhibited the PASMCs' proliferation in PAH mice with less inflammation and reactive oxygen species levels. Moreover, CBD alleviated rodent PAH by recovering mitochondrial energy metabolism, normalizing the hypoxia-induced oxidant stress, reducing the lactate overaccumulation and abnormal glycolysis.

**Conclusions:** Taken together, these findings confirm an important role for CBD in PAH pathobiology.

## Introduction

Pulmonary arterial hypertension (PAH) is a fatal disease associated with the vascular cell dysfunction, hyperproliferation and infiltration of inflammatory macrophages 1, 2. Growing evidence has implicated that mitochondria were involved in the pathogenic mechanism of PAH 3-5. Mitochondrial fission and fusion play critical roles in maintaining functional mitochondria, while cells undergo metabolic or environmental stress 6. Furthermore, PAH is closely associated with the changes of the intracellular oxidant state, many antioxidant factors maintain the vascular homeostasis, such as the nuclear factor E2 related factor 2 (NFE2L2), heme oxygenase 1 (HMOX-1), quinone oxidoreductase 1 (NQO1) and superoxide dismutase (SOD), whose expressions or enzyme activities may partially reflect the oxidant state of the organism 7, 8. Cells under inflammation preferentially use glycolysis as a source of energy, whereas during the resolution phase, they mainly rely on OXPHOS metabolism 9. A shift from oxidative phosphorylation to glycolysis occurs in PAH pulmonary vessels and right ventricle 10, and these two respiratory methods maintain the metabolic balance in cells.

At present, five classes of drugs targeting PAH have been approved by FDA, such as bosentan, sildenafil, riociguat, epoprostenol and selexipag 11. Even with the advances that have been made in PAH treatment, patients still suffer from severe side effects like liver toxicity 12. New drugs with higher efficacy and less side-effects are urgently required.

*Cannabis sativa* is a remarkable plant that contains many valuable components, it consists of 483 known compounds used for medical or recreational purposes, including more than 60 unique compounds known as cannabinoids 13, 14. Apart from the psychoactive constituent delta-9-tetrahydrocannabinol (THC), the non-psychoactive constituents cannabidiol (CBD), tetrahydrocannabivarin (THCV), cannabidivarin (CBDV) and cannflavin A (CFA) have been widely reported to elicit therapeutic effects in analgesia and anti-inflammation in mice 15. In 2018, FDA approved the effect of CBD in reducing seizures related to a rare form of pediatric epilepsy 16. It was reported that CBD ameliorated monocrotaline (MCT)-induced PAH in rats, by improving endothelial functions, normalization of hemostatic alterations and reduction of enhanced leukocyte count that occurred in PAH 17. CBD also showed therapeutic usage in pathological conditions of heart dysfunction and vascular abnormality, by improving both heart and arteries performance along with its anti-inflammatory and antioxidant effects 18, 19, whereas the molecular action of CBD in PAH is still unknown.

In this study, we wanted to analyze the molecular mechanisms underlying the therapeutic effects of CBD for the prevention and treatment of PAH.

## Methods

### Experimental animals

All the experiments were performed in accordance with the NIH guidelines for the Care and Use of Laboratory Animals. All the procedures were approved by the Ethics Committee for Animal Experimentation of China Agricultural University. Male C57BL/6J mice and Sprague-Dawley rats were purchased from Beijing Vital River Laboratory Animal Technology Co., Ltd. China, they were housed in a 12 h light/dark cycle under specific pathogen-free conditions. The Lyz2tm1(cre) Cnr2 knockout mice (C57BL/6J background, with Cnr2 knockout in macrophages) was a generous gift from professor Zhinan Yin, Jinan University. The Cnr2 knockout mice were generated by hybridizing them with a tool mice (Dppa3, C57BL/6J*^-Dppa3em1(IRES-Cre)Smoc^*).

### PAH animal models

Sugen-hypoxia PAH mouse models were induced according to the previously reported study 20, 21.

For the preventive mouse model, male C57BL/6J mice (n = 10 per group) were randomly divided into several groups. Normoxia control (treated with vehicle, 0.1% alcohol, intragastrical (i.g.) injection) and Normoxia CBD (daily treated with 10 mg/kg, i.g.) groups were treated without hypoxia or Sugen. Hypoxia control (treated with 0.1% alcohol, i.g.) and Hypoxia with CBD (daily treated with 10 mg/kg, i.g.) groups were given weekly subcutaneous injection of Sugen (20 mg/kg) in vehicle (0.4% polysorbate 80, 0.5% carboxyl methylcellulose sodium, 0.9% benzyl alcohol) and placed in an animal incubator CJ-DO2 (oxygen concentration maintained between 9% and 11%, Changsha Changjin Technology Co., China) for 21 days. On day 22, animals were anaesthetized with ketamine (100 mg/kg) and xylazine (10 mg/kg), the concentration was gradually increased for about 7 min, the anesthesia depth was monitored with pedal reflex. Right ventricular function was assessed, the hearts, lungs and blood were harvested. The right lung was snap frozen in liquid nitrogen. The left lung was fixed with 4% paraformaldehyde in PBS prior to dehydration and paraffin embedding.

For the therapeutic mouse model, male C57BL/6J mice (n = 10 per group) were given weekly subcutaneous injection of Sugen (20 mg/kg) or vehicle and placed in an animal incubator CJ-DO2 (oxygen concentration maintained between 9% and 11%), allowed to develop PAH from day 0 to 35. Mice in Nomoxia group and Sugen-hypoxia group were received vehicle control (0.1% alcohol, i.g.) from day 22 till day 35. Mice in Normoxia-CBD group and Hypoxia-CBD group were treated with CBD (daily 10 mg/kg, i.g.) from day 22 to day 35. All animals were sacrificed on day 36 after the Sugen-hypoxia inducement. Tissue harvesting was carried out as described above.

The method for Monocrotaline (MCT) induced preventive PAH was described in the previous study 22. Male Sprague Dawley rats (~200 to 250 grams) were randomized into four groups. Control rats were given subcutaneous injection of sterile water as a vehicle control. MCT rats were given 60 mg/kg of MCT by subcutaneous injection and treated with intraperitoneal (i.p.) injections of CBD (daily 10 mg/kg) or vehicle control from day 0 until day 21. All animals were sacrificed on day 22. Right ventricular function was assessed, tissue harvesting was carried out as described above.

### Analysis of right ventricular hypertrophy and right ventricular systolic pressure

The right ventricular hypertrophy (RVH) was measured by calculating the ratio of the weight of right ventricular (RV) to the weight of left ventricular (LV) plus septum as our study described previously 20.

Right ventricular systolic pressure (RVSP) was measured with a micro-pressure transducer (Samba Preclin 420 LP transducer, Samba Sensors, Sweden). It is an invasive test called heart catheterization, the catheter was insert into the right ventricle of the mouse/rat heart along the right carotid artery, the RVSP curve and data were measured and analyzed with the BE-EH4 biological signal system (Beijing Baianji Science and technology company, China) as described previously 23.

### Vascular remodeling analysis

After paraffin embedding and sectioning, the lung slides (4 μm thickness) were stained with hematoxylin and eosin (H.E.) for morphological analysis. To assess the degree of pulmonary arterial remodeling, microscopic images of Elastin van Gieson and α-smooth muscle actin-stained lung sections were captured using the Olympus BX53 microscope (Olympus, Japan). Pulmonary vascular remodeling was quantified by accessing the medial wall thickness and the percentage of muscularization as descripted previously 24-27. To determine the degree of medial wall thickness, < 50 μm and > 50 μm in diameter from each lung were randomly outlined by an observer blinded to mouse group or antibody treatment. The degree of medial wall thickness, expressed as a ratio of medial area to cross sectional area (Media/CSA), were analyzed using Image J. More than 20 vessels with diameters ranging from 25 to 75 μm were counted from non-serial lung sections and categorized as either fully (i.e., with a complete medial coat of muscle), partially (i.e., with only a crescent of muscle) or non-muscularized (i.e., with no apparent muscle) 26.

### Statistical Analysis

All graphs were performed using GraphPad Prism 8.0.2 (GraphPad Software, USA). The data was analyzed by SPSS12.0.1 (SPSS, USA). All results were shown as mean ± SEM. Comparisons of means between 2 groups were performed by unpaired Student's t-test for normally distributed cases. Comparisons of means among three or more groups were performed by one-way or two-way ANOVA (analysis of variance) followed by Bonferroni's multiple comparison test or the Dunnett's method for multiple comparison. All reported *P* values were 2-tailed, with a *P* value of less than 0.05 indicating statistical significance.

Extended Material and Methods section is available in the Supplementary material.

## Results

### CBD inhibited mice PAH-PASMCs proliferation without cytotoxicity

**Figure 1:**
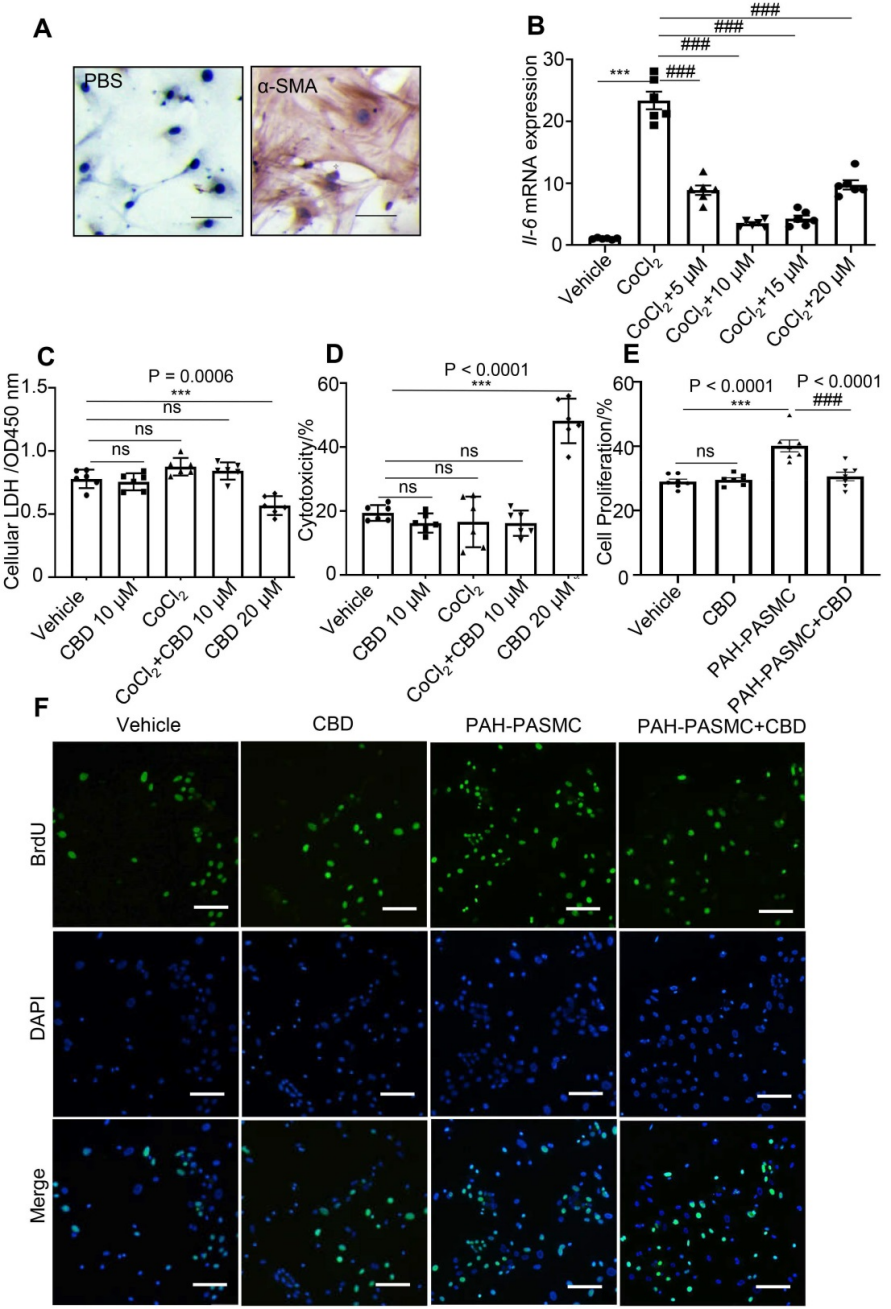
** Inhibition of CBD in mice PASMCs' hyperproliferation without cytotoxicity. A**, Purity of mice PASMCs assessed by immunohistochemistry with the α-SMA antibody. Scale bar = 100 µm **B**, mRNA levels of *Il6* in mice PASMCs, incubation with different concentrations of CBD and/or CoCl_2_ at 200 µM for stimulating the hypoxia condition, n = 6 per group. **C** and **D**, Level of LDH assessed with LDH detection assay in both extracellular (death rate) and intracellular (cell viability) (n = 6 per group). **E**, Quantitative assessment of BrdU antibody to calculate the ratio of PASMCs proliferation. **F**, Immunofluorescence of BrdU positive ratio of the PASMCs (n = 7 per group), the nuclei of cells were stained with 4',6-diamidino-2-phenylindole (DAPI). The results were analyzed by one-way ANOVA followed by Bonferroni's multiple comparison test, *P < 0.05, **P < 0.01, ***P < 0.001 vs. the control group, and ^#^*P* < 0.05, ^##^*P* < 0.01, ^###^*P* < 0.001 vs. the CoCl_2_ group or mice PAH-PASMCs.

To find a potential drug for PAH treatment, several cannabinoid compounds extracted from *Cannabis sativa*, including CBD, CBDV, THCV, CFA, have been screened in mice PASMCs. The purity of the isolated mice PASMCs was confirmed above 90% by immunochemistry (Figure 1A). Our results showed that CBDV, THCV, CFA did not inhibit the hyperproliferation of mice PAH-PASMCs as effectively as CBD, and some of them showed various degrees of cytotoxicity (Figure S1). Therefore, we focused on CBD, which exhibited strong anti-proliferation and less toxicity in mice PASMCs, and it could also reduce the CoCl_2_-induced expression of *Il6* (Figure 1B). Normally, lactate dehydrogenase (LDH) is present in the living cells and leaks out once the cells die, and it can be used for estimating cell viability and cytotoxicity. By using the LDH assay, we found that CBD at 20 μM showed higher cytotoxicity and reduced cell viability in mice PASMCs, while CBD at 10 μM had no effect on the normal mice PASMCs (Figure 1C, 1D). Furthermore, the cell proliferation assay confirmed that CBD at 10 μM could inhibit the hyperproliferation of mice PAH-PASMCs (Figure 1E-F). CBD therefore could inhibit PAH-PASMCs hyperproliferation without any harmful effects on normal PASMCs.

### CBD ameliorated PAH in preventive and theraputic rodent models

To explore the efficacy of CBD in the PAH, we established several rodent PAH models, SU-5416 (Sugen) hypoxia-induced PAH mouse models, which including the preventive model and therapeutic model, and monocrotaline (MCT)-induced PAH rat preventive model. By using a hypoxia-induced PAH preventive mouse model (with CBD treatment at 10 mg/kg and 20 mg/kg, respectively), we found that CBD at 10 mg/kg had the better efficacy to attenuate PAH phenotypes in PAH mice (Figure S2). Based on above results and other reports 28-31, the effective dose of CBD in Sugen hypoxia- and MCT-induced PAH rodent models was established as 10 mg/kg.

**Figure 2:**
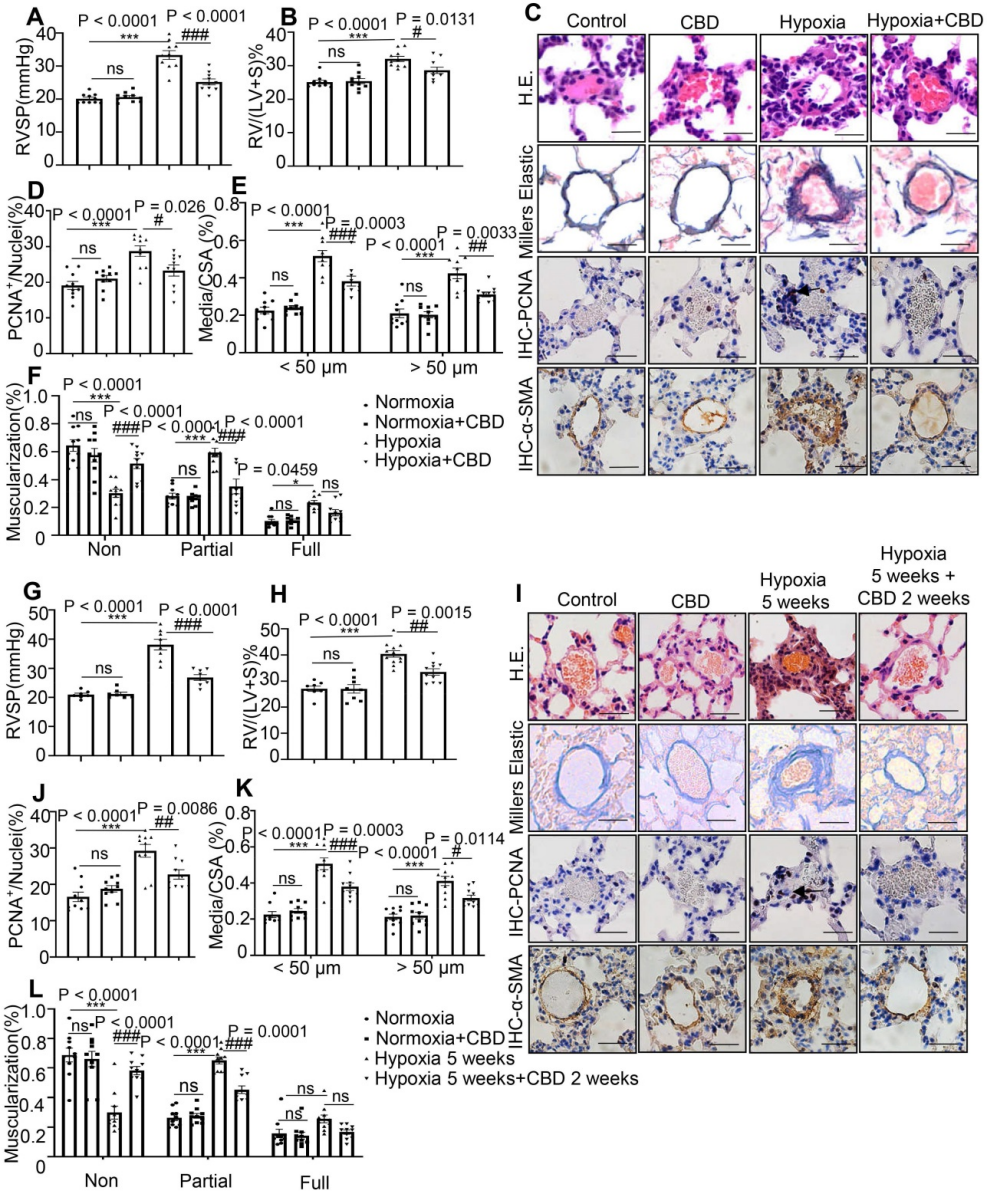
** CBD reversed pathological progression of Sugen-hypoxia induced PAH in both preventive and therapeutic mouse models. A-F**, Data from PAH preventive mice model.** G-L**, Data from PAH therapeutic mice model. **A** and **G**, Assessments of RVSP. **B** and **H**, Assessments of RVH.** C** and **I**, Representative images of pulmonary arteries stained with H&E, and representative images of vascular remodeling in the distal arterioles stained with elastin, immunostained for PCNA and α-SMA. Scale bar = 20 µm. **D-F** and **J-L**, Pulmonary vascular remodeling rate in the Sugen-hypoxia mice, including the quantification of the relative number of PCNA^+^/nuclei, the degree of medial wall thickness as a ratio of total vessel size (Media/CSA), and the proportion of non-, partially-, or fully- muscularized pulmonary arterioles (25 to 75 μm in diameter) from PAH mouse model, n = 10 per group. The results were analyzed by one-way ANOVA followed by Bonferroni's multiple comparison test, *P < 0.05, **P < 0.01, ***P < 0.001 vs. the control group, and ^#^*P* < 0.05, ^##^*P* < 0.01, ^###^*P* < 0.001 vs. the hypoxia group.

In the preventive models, mice or rats that treated with CBD showed attenuated PAH phenotypes, compared with the hypoxia-induced mice and the MCT-induced rats, respectively. These effects included the reductions in right ventricular systolic pressure (RVSP), right ventricular hypertrophy (RVH) (Figure 2A-B, Figure S3A-B), the PAH-related inflammation and the remodeling rates of the pulmonary arteries (Figure 2C-F, Figure S3C-F).

In the therapeutic model, we examined the efficacy of CBD in the established Sugen hypoxia-induced PAH mice, these mice presented with higher RVSP and RVH (Figure 2G-H) as well as extensive pulmonary arterial muscularization (Figure 2I-L). After daily administration of CBD for 2 weeks, the PAH mice showed attenuated lung phenotypes, including the reductions in RVSP, RVH and vascular remodeling rates, compared to saline treated PAH controls (Figure 2G-L).

Next, we compared it with the other compounds that used in the first-line therapy against PAH, such as bosentan and beraprost sodium 32. The results illustrated that all these three compounds could attenuate the elevated RVSP and RVH in the Sugen hypoxia-induced PAH mice. Efficacies among CBD, bosentan and beraprost sodium were very similar, and no significant difference was observed in the prevention of Sugen-hypoxia induced PAH (Figure S4A-B). Taken together, these results demonstrated that CBD could inhibit the development of PAH and its effect was comparable with the first-line PAH drugs in rodent models.

### CBD inhibited the inflammation in PAH mice independent of cannabinoid receptors

**Figure 3:**
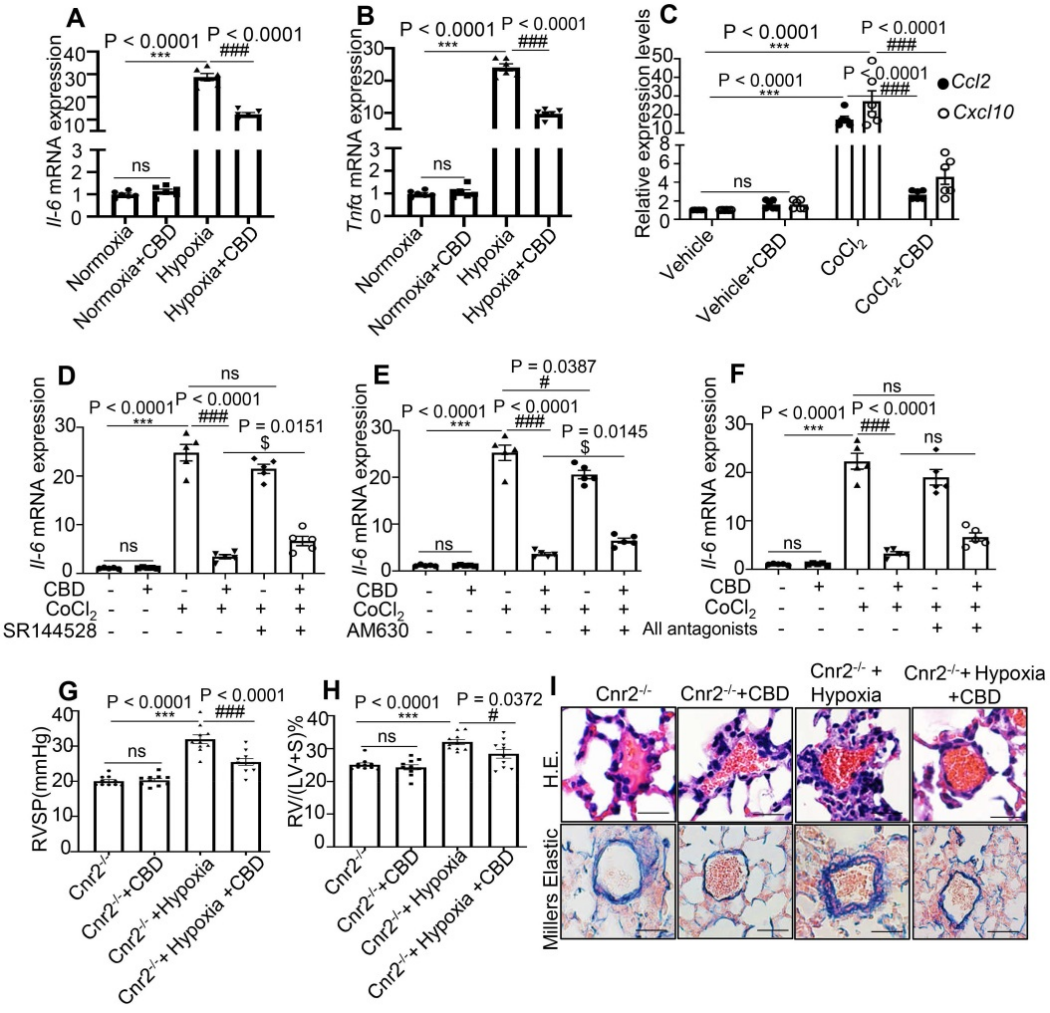
** CBD inhibited inflammation in PAH without canonical cannabinoid receptors involvement. A-B**, mRNA levels of *Il6* and *Tnfα* assessed in the lungs of hypoxia-induced preventive PAH mouse model. **C**, mRNA levels of *Ccl2* and *Cxcl10* assessed in the mice PASMCs with or without CoCl_2_ treatment. **D-F**, mRNA level of *Il6* in mice PASMCs treated with CoCl_2_, and the effect of antagonists of cannabinoid receptors (SR144528, AM630 and both), the concentration of the antagonists was 10 µM, which was equal to the concentration of CBD, the antagonists were pre-treated for 30 min before CBD added, n = 6 per group.** G-H**, Assessments of RVSP, RVH in the PAH Cnr2^-/-^ mice, n = 10 per group. **I**, Representative images of pulmonary arteries stained with H&E and elastin of Cnr2^-/-^ and WT littermate controls with or without Sugen hypoxia-induced or daily CBD-treated, scale bar = 20 µm. The results were analyzed by one-way ANOVA followed by Bonferroni's multiple comparison test, *P < 0.05, **P < 0.01, ***P < 0.001 vs. the control group, and ^#^*P* < 0.05, ^##^*P* < 0.01, ^###^*P* < 0.001 vs. the hypoxia or CoCl_2_ treatment group, ^$^*P* < 0.05 vs. the CoCl_2_ with CBD group.

CBD treatment significantly suppressed the mRNA levels of *Il6* and *Tnfα* in the mouse lungs of Sugen-hypoxia preventive mouse model, as well as the mRNA levels of chemokine *Ccl2* and *Cxcl10* in mice PASMCs (Figure 3A-C). As CBD had been reported to elicit its molecular action via cannabinoid receptor 1 (Cnr1), cannabinoid receptor 2 (Cnr2), transient receptor potential A1 (TRPA1) and peroxisome proliferator-activated receptor (PPARγ) 33, we assessed the expression of *Il6* in mice PASMCs with or without antagonist or inhibitor for cannabinoid receptors, such as Rimonabant (antagonist for Cnr1), SR144528 and AM630 (antagonist for Cnr2), GW9662 (antagonist for PPARγ) and HC030031 (inhibitor for TRPA1). Our results showed that CBD could normalize the increased expression of* Il6* in the CoCl_2_ treated group, whereas the antagonists of Cnr1, TRPA1 and PPARγ could not counteract the anti-inflammatory effects of CBD in mice PASMCs (Figure 3D-F), the expression of *Il6* was increased with Cnr2 antagonists (SR144528, AM630) (in combination with CBD in hypoxia treated PASMCs), compared to the CBD treatment alone under hypoxia condition (Figure 3D-F and Figure S5). To clarify whether the inhibition of CBD on PAH was mainly via Cnr2 signaling pathway, we generated the Cnr2^-/-^ mice. In Sugen-hypoxia induced Cnr2^-/-^ PAH mouse model, we did not observe any significant phenotypical or pathological differences between wild type PAH mice and Cnr2^-/-^ PAH mice. However, CBD could reverse the phenotypes of PAH in both wild type mice and Cnr2^-/-^ mice, including RVSP, RVH, and pathological index of PAH (Figure 3G-I). All these results indicated that Cnr2 was not involved in the inhibition of CBD in PAH development.

### CBD restored mitochondrial function and oxidant stress in mice PAH-PASMCs

**Figure 4:**
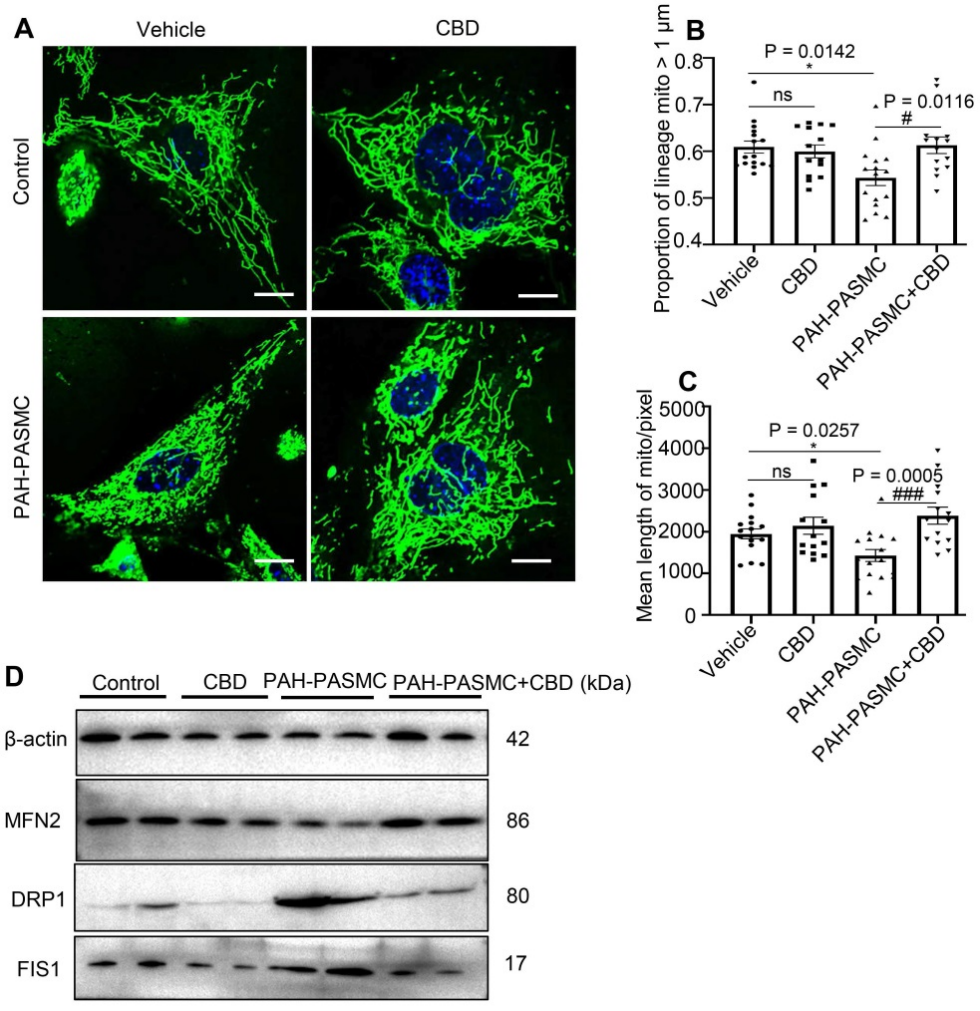
** CBD improved mitochondrial function in mice PAH-PASMCs. A**, Representative images of PASMCs isolated from the control and PAH mice labeled with MitoTracker after the treatment with CBD (10 µM) or the control vehicle for 2 h. Nuclei were counterstained using Hoechst 33342, scale bar = 10 µm. **B** and **C**, Quantification of mitochondrial proportion of lineage that longer than 1 µm (40 pixels, about 1 µm) and mean length of mitochondria in control PASMCs and PAH-PASMCs labeled with Mitotracker after treatment with or without CBD for 2 h, n = 20 per group. **D**, Immunoblotting for DRP1, FIS1, MFN2 proteins in mice PAH-PASMCs with CBD (10 µM) or vehicle for 12 h. All blots were re-probed for β-actin as control. The results were analyzed by one-way ANOVA followed by Bonferroni's multiple comparison test, *P < 0.05, **P < 0.01, ***P < 0.001 vs. the control group, and ^#^*P* < 0.05, ^##^*P* < 0.01, ^###^*P* < 0.001 vs. the mice PAH-PASMCs.

By using MitoTracker, we found that CBD treatment could normalize the malfunctioning mitochondrial networks (i.e., increased proportion of lineage and mean length of mitochondria) in both hypoxia-induced human PASMCs and mice PAH-PASMCs (Figure 4A-C, Figure S6A-C). There are a set of genes that were reported to promote mitochondrial fission, including dynamin-1-like protein (*DRP1*), mitochondrial fission 1 protein (*FIS1*) and optic atrophy type 1 (*OPA1*), and a group of genes for mitochondrial fusion, such as mitofusin 1 (*MFN1*), mitofusin 2 (*MFN2*), and mitochondrial elongation factor (*MIEF1*) 34. We found that CBD treatment significantly reduced the mRNA levels of *DRP1, FIS1, OPA1*, whereas the expressions of *DRP1, FIS1, OPA1* were upregulated in hypoxia-induced human PASMCs (Figure S6D-F), and CBD could also decrease the protein expressions of DRP1 and FIS1 in the mice PAH-PASMCs (Figure 4D). Conversely, CBD upregulated the mRNA of *MFN1, MFN2*, and *MIEF1* in hypoxia-induced human PASMCs (Figure S6G-I) and protein expression of MFN2 in mice PAH-PASMCs (Figure 4D), compared with the group without CBD treatment, respectively. Together, CBD normalized the mitochondria morphology in PASMCs and sustained the cell homeostasis.

**Figure 5:**
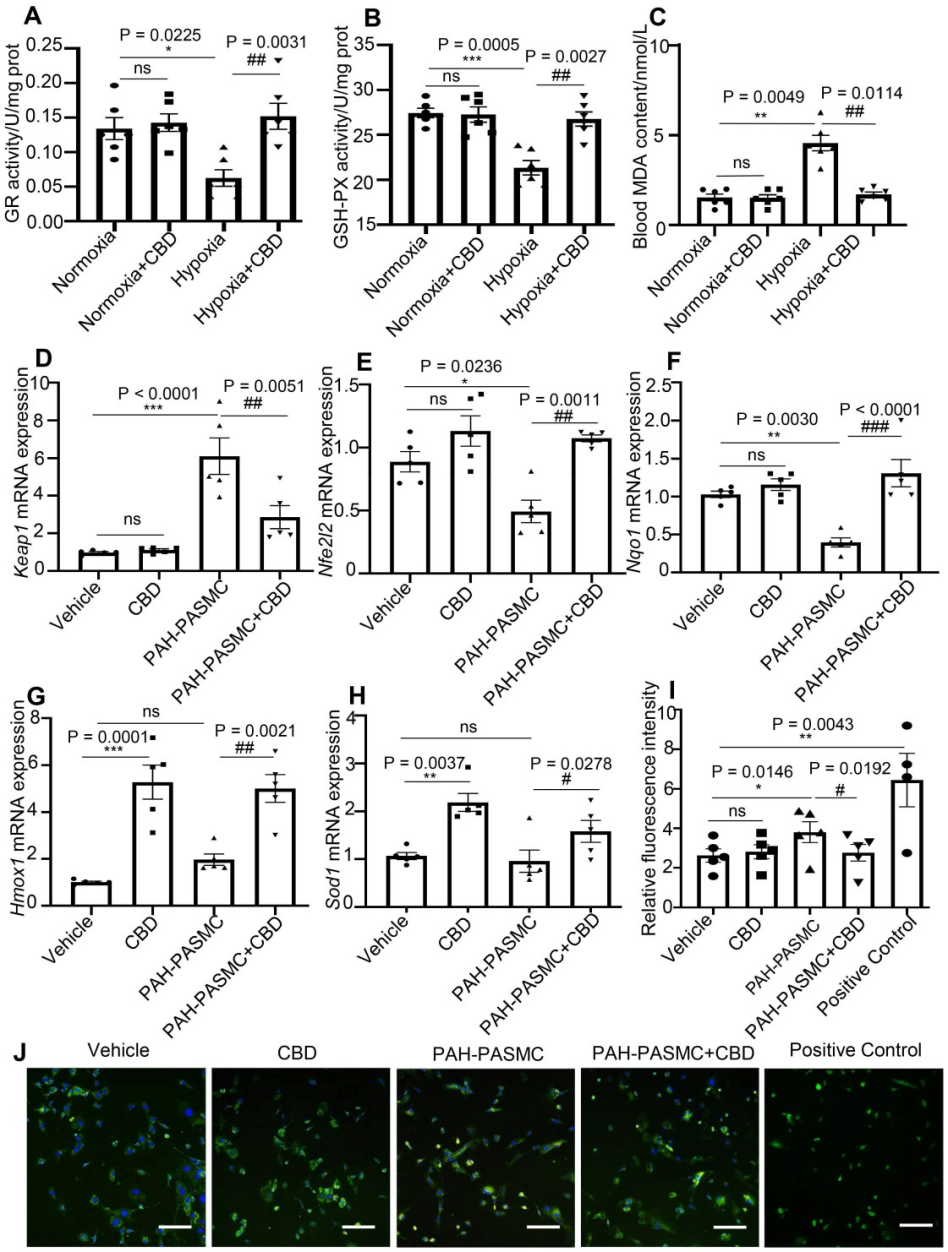
** Analysis of the protection effect of CBD on oxidant stress. A-B**, Quantification of enzyme activities of GR and GSH-PX in the lungs of hypoxia-induced preventive PAH mice, n = 6 per group. **C**, MDA content in the blood of preventive hypoxia-induced PAH mice, n = 6 per group. **D-H**, mRNA levels of *Keap1*, *Nfe2l2*, *Nqo1*, *Hmox1* and *Sod1* quantified using RT-PCR in PASMCs, which were isolated from control and PAH mice after the treatment with CBD (10 µM) or control vehicle, were n = 5 per group. **I**, Quantification of ROS with a fluorescence 96-plate by a fluorescence microplate reader, DCFH-DA fluorescence intensity in PASMCs isolated from control and PAH mice after the treatment with CBD (10 µM) or control vehicle or rosup (provided in the kit as a positive control for ROS) for 2 h, n = 8 per group. **J**, Representative images of ROS fluorescence in PASMCs isolated from control and PAH mice, assessed by laser scanning microscope. Nuclei was counterstained using DAPI, n≥7 per group. Scale bar = 100 µm. The results were analyzed by one-way ANOVA followed by Bonferroni's multiple comparison test, *P < 0.05, **P < 0.01, ***P < 0.001 vs. the control group, and^ #^*P* < 0.05, ^##^*P* < 0.01, ^###^*P* < 0.001 vs. the mice PAH-PASMCs group.

**Figure 6:**
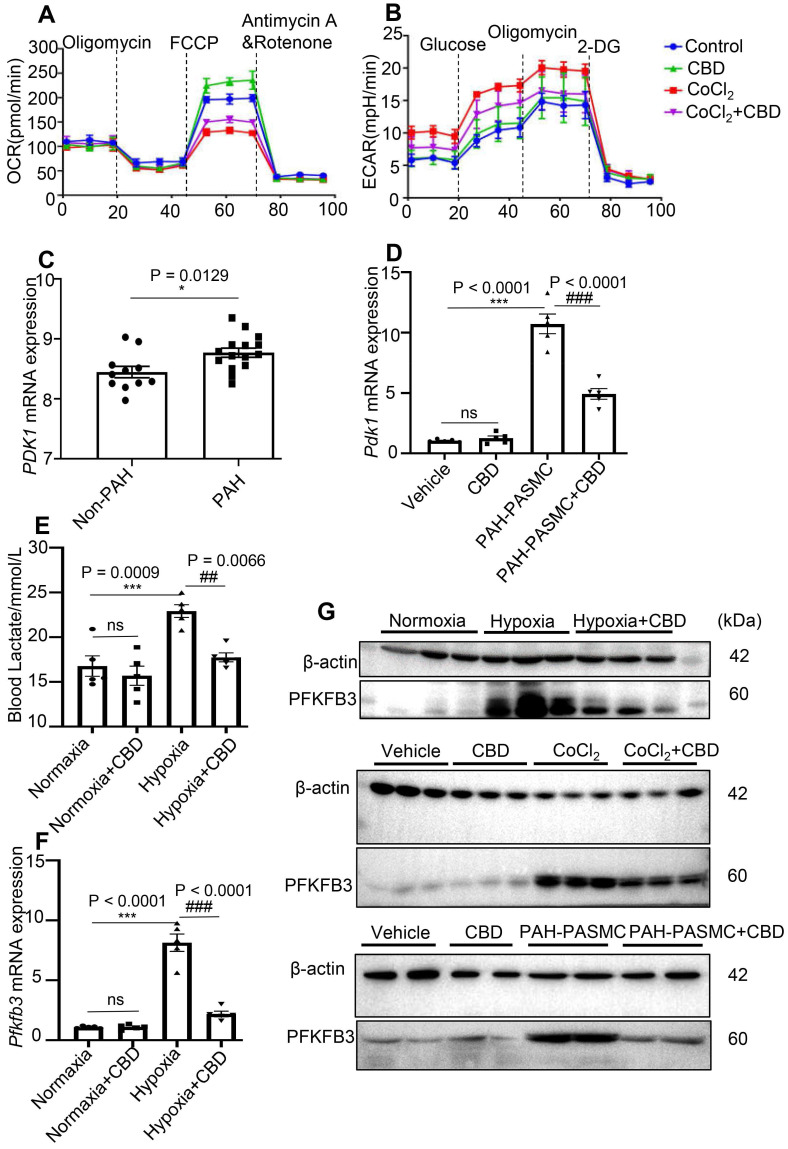
** CBD prevented the transition from oxidative phosphorylation to glycolysis in PAH. A-B**, Quantification of the mitochondrial OCR and ECAR of control human PASMCs and hypoxia-induced human PASMCs after treatment with CBD or the control vehicle for 12 h, n = 5 per group. **C**, Gene expression of *PDK1* in the lungs of patients with PAH (n = 15) and control subjects (n = 11), analyzed by an unpaired two-tailed Student's t-test. **D**, Quantification of *Pdk1* in PASMCs isolated from control and PAH mice after the treatment with or without CBD for 12 h, n = 5 per group. **E**, Contents of lactate in the blood of hypoxia-induced preventive PAH mice detected with the lactate assay, n = 6 per group. **F**, mRNA level of *Pfkfb3* quantified in PAH mice lungs with or without CBD treatment, n = 5 per group. **G**, Immunoblotting for PFKFB3 protein in hypoxia-induced preventive PAH mice lungs (up), control human PASMCs and hypoxia-induced human PASMCs after the treatment with or without CBD (middle) and PASMCs isolated from control and PAH mice after the treatment with CBD (below). All blots were re-probed for β-actin as control. The results were analyzed by one-way ANOVA followed by Bonferroni's multiple comparison test, *P < 0.05, **P < 0.01, ***P < 0.001 vs. the control group, and ^#^*P* < 0.05, ^##^*P* < 0.01, ^###^*P* < 0.001 vs. the hypoxia or mice PAH-PASMCs group.

CBD had been reported to affect redox balance by modifying both oxidants and antioxidants 35, 36. Glutathione reductase (GR) and glutathione peroxidase (GSH) have been reported to protect the structure and function of cell membranes from peroxides damage 37. We observed that CBD could normalize the enzyme activities of GR and GSH in the lungs of PAH mice (Figure 5A-B). The content of malondialdehyde (MDA), a product of peroxidation, was elevated in the blood of PAH mice, and CBD treatment significantly reduce this elevation (Figure 5C). Furthermore, to assess the antioxidant functions of CBD, the mRNA expressions of some canonical genes involved in oxidative stress were examined. We observed that CBD significantly reduced the mRNA level of Kelch-like ECH-associated protein 1 (*Keap1*) in mice PAH-PASMCs. CBD could also upregulate the expressions of *Nrf2* (*Nfe2l2*) (a master regulator of cellular response against oxidative stress), and its downstream genes, such as *Nqo1*, *Hmox1*, and *Sod1* (Figure 5D-H). Moreover, we observed that CBD significantly reduced the high level of cytosolic reactive oxygen species (ROS) in hypoxia-induced human PASMCs and mice PAH-PASMCs, compared with the control PASMCs (Figure 5I-J, Figure S7A-B). Next, by using Seahorse XF24-3 apparatus, we evaluated the effects of CBD on the O_2_ consumption rate (OCR) and extracellular acidification rate (ECAR) of human PASMCs. We observed a significant decrease in ATP, maximal respiration, and OCR/ECAR ratio in hypoxia-induced human PASMCs compared with the control PASMCs, whereas CBD treatment significantly improved these parameters (Figure 6A-B and Figure S8A-D). These results suggested that CBD treatment sustained the normal oxidant state of PAH-PASMCs and improved mitochondrial energy metabolism in PASMCs.

### CBD inhibited glycolysis and reversed the abnormal metabolism in PAH

Oxidative phosphorylation and glycolysis were the main reactions of ATP production, and it had been reported that inhibition of mitochondrial respiration can enhance glycolysis and vice versa 38.We found that CBD treatment reduced the hypoxia-increased glycolysis in human PASMCs (Figure S8E-F). To verify our findings on glycolysis in vitro and in vivo, we re-analyzed the RNA-seq data of PAH patients from the public database (No.GSE113439) 39, we found an increased expression of pyruvate dehydrogenase kinase isozyme 1 (*PDK1,* a key enzyme in glycolysis) in the lungs from 15 PAH patients (Figure 6C). Additionally, we observed that CBD significantly reduced the mRNA level of *Pdk1*, which was elevated in mice PAH-PASMCs (Figure 6D). Recently, lactate overaccumulation in PAH patients had been reported 40. We found that lactate accumulation in the blood of hypoxia-induced PAH mice was reduced by CBD treatment (Figure 6E). Next, we observed that CBD could normalize the high expression level of glucose transporter 1 (*Glut1*) in the lungs of PAH mice (Figure S8G). It had been proved that the shift from oxidative phosphorylation to glycolysis was due to the activation of 6-phosphofructo-2-kinase/fructose-2,6-biphosphatase 3 (PFKFB3) in the hyperproliferation of PASMCs in PAH 41. We observed that CBD could reduce the elevated mRNA level of *Pfkfb3* in the lungs of hypoxia-induced PAH mice and mice PAH-PASMCs (Figure 6F, Figure S8H), PFKFB3 protein expression pattern was similar in both lungs of PAH mice and in vitro PASMCs (Figure 6G). Taken together, our results demonstrated that CBD could improve mitochondrial functions and inhibit the abnormal glycolysis in PAH.

## Discussion

In this study, we demonstrated the preventive and therapeutic effects of CBD in two conventional PAH rodent models. Mechanically, CBD could inhibit the hyperproliferation of PAH-PASMCs, recover the dysfunctional mitochondria in hypoxia condition, normalize the intracellular oxidant stress, and prevent the lactate overaccumulation and the excessive glycolysis, which eventually ameliorated PAH. These mechanistic findings provided an insight for a novel therapeutic potential for CBD in PAH.

In this study, we demonstrated that CBD treatment not only showed highly effective for the prevention of PAH mouse and rat models, but could also reverse the established disease in PAH mice as well. The inhibitory roles of CBD for the elevated RVSP, RVH as well as the rate of vascular remodeling were remarkable. Based on other reports and the FDA's recommendations about the use of CBD 17, the toxicity of CBD in this study was very low. Although the targets or affected signaling pathways by CBD, bosentan and beraprost sodium are different, CBD presented with comparable efficacy with these first-line PAH drugs. Thus, CBD had the major potential to become a PAH drug, while future clinical investigation for the efficacy of CBD in PAH patients is of high importance.

CBD had been reported to act through several receptors like Cnr1, Cnr2, TRPA1 and PPARγ 42, 43. In this study, we found that CBD exerted its anti-inflammatory effects in PASMCs independent of Cnr1, TRPA1 and PPARγ signaling pathways. The inhibitors of Cnr2 receptor had the ability to partially counteract the anti-inflammatory effects of CBD in vitro, but no significant phenotypical and pathological differences were observed between wild type PAH and Cnr2^-/-^ PAH mice. Moreover, CBD could still reverse the phenotypes of PAH both in WT and Cnr2^-/-^ mice, which strongly implicated that the inhibitory effects of CBD on PAH in vivo was Cnr2-independent. The reasons for this discrepancy are not fully understood yet, we could speculate it may be due to: On the one hand, in vitro models are routinely used in biological fields to find mainly signaling targets, while in vivo animal models are more relevant to the physiological and pathological conditions. On the other hand, the affinity between Cnr2 and CBD is very low 44, and Cnr2 is not the key mediator for CBD action in PAH. Therefore, CBD labeling and CBD-specific receptor searching experiments are currently undergoing in our lab. Due to the structural limitation of CBD itself, the biotin labeled-CBD at several sites of carbon-skeleton eliminated the chemical properties of CBD. New technologies and methods, such as isotope labelling and single molecule imaging, might be the feasible solutions.

Generally, the states of fission and fusion maintain a dynamic balance of mitochondria 45. In our study, we found that CBD could downregulate the fission mediators and upregulate the fusion mediators in PASMCs. Accordingly, CBD treatment increased the mitochondrial networks in hypoxia-induced human PASMCs and mice PAH-PASMCs, which resulted in an increased integrity of mitochondrial structure. Whereas how CBD becomes involved in the gene regulation of the fusion/fission balance, needs to be further studied.

We also observed that CBD could regulate the genes related to oxidative stress (*Hmox1, Sod1*) in the normal mice PASMCs. Previous studies have also reported the strong antioxidant capacity of CBD, and CBD can upregulate *Hmox1*, activate the NRF2-keap1 pathway and normalize the cell functions under the oxidant stress 36. Based on these results, we could speculate that CBD had the efficacy to maintain the mitochondrial homeostasis in both patho-/physiological conditions.

The vasodilatory effects of CBD in mice had been reported 46, and this group recently demonstrated that CBD ameliorated MCT-induced PAH in rats by improving endothelial function, normalization of hemostatic alterations and reduction of enhanced leukocyte count determined in PAH 17, whereas no direct target for CBD was reported in their PAH prevention study. Consistent with our study, they also suggested that the effects of CBD in PAH offered further potential pharmacological opportunities for CBD in vascular obstructive pulmonary diseases.

CBD is a non-psychoactive drug with less side-effects 47, which could be further considered as a candidate drug for PAH. CBD may offer a possibility for combined therapy with other first-line medicines for PAH, but a large-scale clinical investigation on CBD in PAH patients is needed.

## Conclusions

We demonstrated that CBD inhibited the hyperproliferation of PASMCs, recovered the function of mitochondria, alleviated the oxidant stress in PASMCs and inhibited the excessive glycolysis, accompanied by metabolic improvement (Graphical abstract). Consistently, CBD successfully ameliorated hypoxia-induced PAH in mice and MCT-induced PAH in rats. Taken together, our study strongly suggested that CBD might provide a promising novel potential for the treatment of PAH.

## Supplementary Material

Supplementary figures and tables.

## Abbreviation

PAH: pulmonary arterial hypertension
PASMCs: pulmonary artery smooth muscle cells
CBD: cannabidiol
ROS: reactive oxygen species
Nfe2l2: nuclear factor E2 related factor 2
Hmox1: heme oxygenase 1
Sod: superoxide dismutase
Nqo1: quinone oxidoreductase 1
Cnr: cannabinoid receptor
TRPA1: transient receptor potential A1
PPARγ: peroxisome proliferator-activated receptor
Sugen: vascular endothelial growth factor receptor blocker SU-5416
MCT: monocrotaline
RVSP: right ventricular systolic pressure
RVH: right ventricular hypertrophy
IHC: immunohistochemistry
PFKFB3: 6-phosphofructo-2-kinase/fructose-2,6-biphosphatase 3
OCR: O_2_ consumption rate
ECAR: extracellular acidification rate
DRP1: dynamin-1-like protein
FIS1: mitochondrial fission 1
OPA1: optic atrophy type 1
MFN1/2: mitofusin 1/2
MIEF1: mitochondrial elongation factor 1
GR: glutathione reductase
GSH: glutathione peroxidase
MDA: malondialdehyde
Keap1: Kelch-like ECH-associated protein 1
Glut1: glucose transporter 1
Pdk1: pyruvate dehydrogenase kinase 1

## References

[1] Chen S, Yan D, Qiu A (2020). The role of macrophages in pulmonary hypertension: Pathogenesis and targeting. Int Immunopharmacol.

[2] Sun L, Lin P, Chen Y, Yu H, Ren S, Wang J (2020). miR-182-3p/Myadm contribute to pulmonary artery hypertension vascular remodeling via a KLF4/p21-dependent mechanism. Theranostics.

[3] Paulin R, Michelakis ED (2014). The metabolic theory of pulmonary arterial hypertension. Circulation research.

[4] Sutendra G, Dromparis P, Bonnet S, Haromy A, McMurtry MS, Bleackley RC (2011). Pyruvate dehydrogenase inhibition by the inflammatory cytokine TNFalpha contributes to the pathogenesis of pulmonary arterial hypertension. Journal of molecular medicine (Berlin, Germany).

[5] Elinoff JM, Mazer AJ, Cai R, Lu M, Graninger G, Harper B Meta-analysis of blood genome-wide expression profiling studies in pulmonary arterial hypertension. 2020; 318: L98-L111.

[6] Liu YJ, McIntyre RL, Janssens GE, Houtkooper RH (2020). Mitochondrial fission and fusion: A dynamic role in aging and potential target for age-related disease. Mech Ageing Dev.

[7] Zhang T, Liang X, Shi L, Wang L, Chen J, Kang C (2013). Estrogen receptor and PI3K/Akt signaling pathway involvement in S-(-)equol-induced activation of Nrf2/ARE in endothelial cells. PLoS One.

[8] Tabima DM, Frizzell S, Gladwin MT (2012). Reactive oxygen and nitrogen species in pulmonary hypertension. Free Radic Biol Med.

[9] Puleston DJ, Villa M, Pearce EL (2017). Ancillary Activity: Beyond Core Metabolism in Immune Cells. Cell metabolism.

[10] Culley MK, Chan SY (2018). Mitochondrial metabolism in pulmonary hypertension: beyond mountains there are mountains. The Journal of Clinical Investigation.

[11] Lau EMT, Giannoulatou E, Celermajer DS, Humbert M (2017). Epidemiology and treatment of pulmonary arterial hypertension. Nat Rev Cardiol.

[12] Barnes H, Yeoh H, Fothergill T, Burns A, Humbert M, Williams TJTCdosr Prostacyclin for pulmonary arterial hypertension. 2019; 5: CD012785.

[13] Navarro G, Varani K, Lillo A, Vincenzi F, Rivas-Santisteban R, Raïch I (2020). Pharmacological data of cannabidiol- and cannabigerol-type phytocannabinoids acting on cannabinoid CB1, CB2 and CB1/CB2 heteromer receptors. Pharmacological Research.

[14] Delgado-Povedano MM, Sánchez-Carnerero Callado C, Priego-Capote F, Ferreiro-Vera C (2020). Untargeted characterization of extracts from Cannabis sativa L. cultivars by gas and liquid chromatography coupled to mass spectrometry in high resolution mode. Talanta.

[15] Grotenhermen F (2004). Pharmacology of cannabinoids. Neuro Endocrinol Lett.

[16] Samanta D (2019). Cannabidiol: A Review of Clinical Efficacy and Safety in Epilepsy. Pediatric neurology.

[17] Sadowska O, Baranowska-Kuczko M, Gromotowicz-Poplawska A, Biernacki M, Kicman A, Malinowska B (2020). Cannabidiol Ameliorates Monocrotaline-Induced Pulmonary Hypertension in Rats. Int J Mol Sci.

[18] Elsaid S, Kloiber S, Le Foll B (2019). Chapter Two - Effects of cannabidiol (CBD) in neuropsychiatric disorders: A review of pre-clinical and clinical findings. In: Rahman S, editor. Progress in Molecular Biology and Translational Science: Academic Press.

[19] Rajesh M, Mukhopadhyay P, Batkai S, Patel V, Saito K, Matsumoto S (2010). Cannabidiol attenuates cardiac dysfunction, oxidative stress, fibrosis, and inflammatory and cell death signaling pathways in diabetic cardiomyopathy. J Am Coll Cardiol.

[20] Zhang J, Lu X, Liu M, Fan H, Zheng H, Zhang S (2019). Melatonin inhibits inflammasome-associated activation of endothelium and macrophages attenuating pulmonary arterial hypertension. Cardiovasc Res.

[21] Ciuclan L, Bonneau O, Hussey M, Duggan N, Holmes AM, Good R (2011). A novel murine model of severe pulmonary arterial hypertension. Am J Respir Crit Care Med.

[22] Long L, Ormiston ML, Yang X, Southwood M, Graf S, Machado RD (2015). Selective enhancement of endothelial BMPR-II with BMP9 reverses pulmonary arterial hypertension. Nat Med.

[23] Chen WC, Park SH, Hoffman C, Philip C, Robinson L, West J Right ventricular systolic pressure measurements in combination with harvest of lung and immune tissue samples in mice. J Vis Exp. 2013: e50023.

[24] Schermuly RT, Dony E, Ghofrani HA, Pullamsetti S, Savai R, Roth M (2005). Reversal of experimental pulmonary hypertension by PDGF inhibition. J Clin Invest.

[25] Luo Y, Teng X, Zhang L, Chen J, Liu Z, Chen X (2019). CD146-HIF-1α hypoxic reprogramming drives vascular remodeling and pulmonary arterial hypertension. Nat Commun.

[26] Lawrie A, Hameed AG, Chamberlain J, Arnold N, Kennerley A, Hopkinson K (2011). Paigen diet-fed apolipoprotein E knockout mice develop severe pulmonary hypertension in an interleukin-1-dependent manner. Am J Pathol.

[27] Hameed AG, Arnold ND, Chamberlain J, Pickworth JA, Paiva C, Dawson S (2012). Inhibition of tumor necrosis factor-related apoptosis-inducing ligand (TRAIL) reverses experimental pulmonary hypertension. J Exp Med.

[28] Moreira FA, Aguiar DC, Guimaraes FS (2006). Anxiolytic-like effect of cannabidiol in the rat Vogel conflict test. Prog Neuropsychopharmacol Biol Psychiatry.

[29] Zieba J, Sinclair D, Sebree T, Bonn-Miller M, Gutterman D, Siegel S (2019). Cannabidiol (CBD) reduces anxiety-related behavior in mice via an FMRP-independent mechanism. Pharmacol Biochem Behav.

[30] Kaplan JS, Stella N, Catterall WA, Westenbroek RE (2017). Cannabidiol attenuates seizures and social deficits in a mouse model of Dravet syndrome. Proc Natl Acad Sci U S A.

[31] Carvalho RK, Santos ML, Souza MR, Rocha TL, Guimaraes FS, Anselmo-Franci JA (2018). Chronic exposure to cannabidiol induces reproductive toxicity in male Swiss mice. J Appl Toxicol.

[32] Hoeper MM, Taha N, Bekjarova A, Gatzke R, Spiekerkoetter E Bosentan treatment in patients with primary pulmonary hypertension receiving nonparenteral prostanoids. 2003; 22: 330-4.

[33] Mechoulam R, Peters M, Murillo-Rodriguez E, Hanus LO (2007). Cannabidiol-recent advances. Chemistry & biodiversity.

[34] Kurosawa R, Satoh K, Kikuchi N, Kikuchi H, Saigusa D, Al-Mamun ME (2019). Identification of Celastramycin as a Novel Therapeutic Agent for Pulmonary Arterial Hypertension. Circulation research.

[35] Atalay S, Jarocka-Karpowicz I, Skrzydlewska E (2019). Antioxidative and Anti-Inflammatory Properties of Cannabidiol. Antioxidants (Basel, Switzerland).

[36] Casares L, Garcia V, Garrido-Rodriguez M, Millan E, Collado JA, Garcia-Martin A (2020). Cannabidiol induces antioxidant pathways in keratinocytes by targeting BACH1. Redox Biol.

[37] Koskenkorva-Frank TS, Weiss G, Koppenol WH, Burckhardt S (2013). The complex interplay of iron metabolism, reactive oxygen species, and reactive nitrogen species: insights into the potential of various iron therapies to induce oxidative and nitrosative stress. Free Radic Biol Med.

[38] Yetkin-Arik B, Vogels IMC, Nowak-Sliwinska P, Weiss A, Houtkooper RH, Van Noorden CJF (2019). The role of glycolysis and mitochondrial respiration in the formation and functioning of endothelial tip cells during angiogenesis. Sci Rep.

[39] Mura M, Cecchini MJ, Joseph M, Granton JT (2019). Osteopontin lung gene expression is a marker of disease severity in pulmonary arterial hypertension. Respirology (Carlton, Vic).

[40] Zhang R, Jing ZC (2016). Energetic Metabolic Roles in Pulmonary Arterial Hypertension and Right Ventricular Remodeling. Current pharmaceutical design.

[41] Kovacs L, Cao Y, Han W, Meadows L, Kovacs-Kasa A, Kondrikov D (2019). PFKFB3 in Smooth Muscle Promotes Vascular Remodeling in Pulmonary Arterial Hypertension. Am J Respir Crit Care Med.

[42] Burstein S (2015). Cannabidiol (CBD) and its analogs: a review of their effects on inflammation. Bioorganic & Medicinal Chemistry.

[43] Turcotte C, Blanchet M-R, Laviolette M, Flamand N (2016). The CB2 receptor and its role as a regulator of inflammation. Cellular and Molecular Life Sciences.

[44] Showalter VM, Compton DR, Martin BR, Abood ME (1996). Evaluation of binding in a transfected cell line expressing a peripheral cannabinoid receptor (CB2): identification of cannabinoid receptor subtype selective ligands. The Journal of pharmacology and experimental therapeutics.

[45] Youle RJ, van der Bliek AM (2012). Mitochondrial fission, fusion, and stress. Science (New York, NY).

[46] Baranowska-Kuczko M, Kozłowska H, Kloza M, Sadowska O, Kozłowski M, Kusaczuk M (2020). Vasodilatory effects of cannabidiol in human pulmonary and rat small mesenteric arteries: modification by hypertension and the potential pharmacological opportunities. Journal of hypertension.

[47] Pickrell WO, Robertson NP (2017). Cannabidiol as a treatment for epilepsy. Journal of neurology.

