# Supplementary material for: Cannabidiol attenuates pulmonary arterial hypertension by improving vascular smooth muscle cells mitochondrial function
Source: Theranostics. 2021 Mar 11;11(11):5267–78. doi: 10.7150/thno.55571 (PMC8039951; doi:10.7150/thno.55571)
Supplement: Supplementary file 1 — Supplementary figures and tables. [file thnov11p5267s1.pdf]

SUPPLEMENTAL MATERIAL

Cannabidiol attenuates pulmonary arterial hypertension by improving vascular smooth muscle cells mitochondrial function

Xiaohui Lu*[1], Jingyuan Zhang*[1], Huijiao Liu[1], Wenqiang Ma[1], Leo Yu[2], Xin Tan[3], Shubin Wang[3], Fazheng Ren[4], Xiru Li[5], Xiangdong Li[1, 4, 6 #]

Running title: Cannabidiol and PAH

[1]State Key Laboratory of Agrobiotechnology, College of Biological Sciences, China Agricultural University, Beijing 100193, China.

[2] Yunnan Hempmon Pharmaceuticals Co. Ltd., Beijing, 100010, China.

[3] Hanma Investment Group Co., Ltd., Beijing, 100010, China.

[4] Department of Nutrition and Health, China Agricultural University, Beijing 100193, China.

[5] Department of Surgery, Chinese PLA General Hospital, Beijing, 100071, China.

[6] Department of Reproduction and Gynecological Endocrinology, Medical University of Bialystok, Bialystok, Poland.

[*] Xiaohui Lu, Jingyuan Zhang contributed equally to this work.

[#]Correspondence: Xiangdong Li, State Key Laboratory of Agrobiotechnology, College of Biological Sciences, China Agricultural University, Beijing 100193, China, xiangdongli68@126.com, 86-10-62734389 (Tel/Fax).

Supplemental Materials

Methods

Cell culture and chemicals

High-purity of CBD, THCV, CBDV, and CFA powders (99.8%) were isolated from *Cannabis sativa* (hemp) and provided by Hanyi Biotechnology Beijing Co., Ltd, China, ethanol or DMSO dissolved. Vascular endothelial growth factor receptor blocker SU-5416 (Sugen) and monocrotaline (MCT) were purchased from Sigma, USA. Rimonabant (antagonist of cannabinoid receptor 1, Cnr1, ethanol dissolved), SR144528 (antagonist of Cnr2, DMSO dissolved), AM630 (antagonist of Cnr2, DMSO dissolved), HC030031 (channel blocker of transient receptor potential A1, TRPA1, DMSO dissolved), GW9662 (antagonist of peroxisome proliferator-activated receptor, PPARγ, DMSO dissolved) were purchased from Sigma, USA. $CoCl_2$ (used for stimulating the hypoxia condition in cells), polysorbate 80, carboxyl methylcellulose sodium and benzyl alcohol were purchased from Sigma, USA. Ketamine and xylazine were purchased from Aladdin, China. Bosentan was purchased from Aladdin, China. Beraprost Sodium was purchased from Sigma, USA.

The human PASMC cell line (No. BNCC340087, passage number: P4-6) was purchased from Beina biology, China, authorized by ATCC. The cells were cultured in Dulbecco's Modified Eagle's Medium-high glucose (Sigma, USA) according to the supplier's instructions.

Immunohistochemistry

For assessment of pulmonary arteriolar muscularization, sections of fixed mouse lung tissue (4 μm) were labeled with monoclonal mouse-anti-mouse/rat/human smooth muscle α-smooth muscle actin (SC-53142, Santa Cruz, USA), followed by mouse enhanced polymer method detection kit (PV-9002, ZSGB-Bio, China). To detect the proliferation of PASMCs, the lung tissue sections were also stained with monoclonal mouse-anti-mouse/rat/human proliferating cell nuclear antigen (PCNA) (SC-56, Santa Cruz, USA). Microscopic images were captured using the Olympus BX53 microscope (Olympus, Japan).

Isolation of mice pulmonary artery smooth muscle cells

Mice pulmonary artery smooth muscle cells (PASMCs) were isolated from wild type or PAH mice and cultured by using a modified method described previously [1]. The purity of PASMCs was checked by immunohistochemistry (IHC) analyses with antibody against α-smooth muscle actin (1:1000; Santa Cruz, USA). The purity of mouse PASMCs was ≥90%, which was qualified for further study.

Detection of cellular reactive oxygen species

Two sets of experiments were carried out to detect cellular reactive oxygen species (ROS) according to the manufacturer's instructions of the Reactive Oxygen Species assay kit (Beyotime, China). The first set was to plate the PASMCs onto the coverslips for 24-well plate, the cells were treated with or without 200 μM $CoCl_2$ and 10 μM CBD or vehicle for 2 h, and the images of ROS staining of cells were captured with fluorescence microscope (Nikon, Japan). The density of fluorescence was normalized with 4',6-diamidino-2-phenylindole (DAPI), which was used to detect cell nucleus. The second set was to seed PASMCs onto the 96-well black plate with a transparent flat

bottom. After treating with or without 200 μM $CoCl_2$ and 10 μM CBD or vehicle for 2 h, the cells were stained with ROS assay kit and the fluorescence of each well was quantified by Fluorescence microplate reader (BioTek, USA).

Antioxidative enzyme activities

Determination of glutathione reductases (GR) activity in the blood of mice was carried out using the GR assay kit (Nanjing Jiancheng, China) by measuring the rate of NADPH oxidation to $NADP^+$. GR catalyzes the reduction of glutathione (Oxidized) (GSSG) to its reduced form glutathione peroxidase (GSH), which is accompanied by NADPH oxidation. The oxidation of NADPH causes a decrease in absorbance at 340 nm, and is directly proportional to the GR activity in the sample. GR activity was expressed in nmol/mg protein/min (nmol of NADPH oxidized to $NADP^+$ by the enzyme during 1 min per mg of protein), and the results were detected by the Microplate reader (Power Wave XS2) (BioTek, USA)

The enzyme activities of GSH of mice whole blood were determined by GSH assay kit (Colorimetric method, absorbance values at 412 nm) (Nanjing Jiancheng, China). Glutathione peroxidase (GSH-PX) can promote the reaction of hydrogen peroxide (H_2O_2) with reduced GSH to produce H_2O_2 oxidative GSSG. The activity of the GSH-PX can be expressed by the speed of its enzymatic reaction, and the activity of the enzyme can be obtained by measuring the consumption of reduced glutathione in the enzymatic reaction. GSH reacts with dithiodinitrobenzoic acid to produce 5-dithiodinitrobenzoic acid anion which presents a relatively stable yellow color. The amount of GSH can be calculated by measuring its absorbance at 421 nm. GSH activity was expressed in nmol/mg of protein/min (nmol of NADPH oxidized to $NADP^+$ by the enzyme during 1 min per mg of protein).

The content of malondialdehyde (MDA) were measured by MDA assay kit (Nanjing Jiancheng, China) according to the manufacturer's instructions. Briefly, 250 μL of 15% TCA and 250 μL of 0.37% TBA were added to 125 μL of homogenate and the samples were vortexed and incubated at 100 °C for 10 min. Next, the mixtures were centrifuged at 12,000 rpm for 5 min at 4 °C. The collected supernatant was again centrifuged, and absorbance was measured at a wavelength $\lambda = 532$ nm. Concentration of MDA in homogenates was calculated using a standard curve prepared with 25 μM TEP and was expressed in nmol of MDA per L of the blood of mice, and the results were detected by the Microplate reader (Power Wave XS2) (BioTek, USA).

Cell death and viability

Cell viability was measured with CCK8 assay (Beyotime, China) according to the manufacturer's instructions.

Cytotoxicity of CBD was assessed by detecting the release of lactate dehydrogenase (LDH) into cell incubation media according to the manufacturer's instructions (Beyotime, China). Normally, the LDH is present in the living cells and leaks out of the cells after the cell dead. The content of the extracellular LDH is used to estimate the cell death rate, the content of the intracellular LDH to estimate the proportion of normal cells. When the LDH content in the cell and in the cell culture fluid were detected spontaneously, we can obtain the relative ratio of live cells and dead cells.

Cell proliferation was assessed by BrdU assay. Briefly, cells were incubated with 10 μM BrdU (Aladdin, China) for 20 h and fixed with 4% paraformaldehyde for 30 min at room temperature. After treated with 0.1% Triton-X100 for 15 min, cells were incubated with 2 M HCl for 30 min and

0.1 M sodium borate buffer at pH 8.5 for 10 min. Next, cells were blocked with 5% bovine serum albumin and anti-BrdU antibody (Abclonal, China) at a 1:200 dilution at 4℃ overnight. The secondary antibody Goat anti-Mouse IgG (H+L) (ZSGB-BIO, China) was applied for 1 h at room temperature. The nucleus of cells was stained with DAPI. Cell fluorescence images were captured with a fluorescence microscope (Nikon, Japan) and quantified using Image J.

Mitochondrial morphology detection

Mice and/or Human PASMCs were treated with/without 200 μM $CoCl_2$ and/or 10 μM CBD for 2 h, then detected with MitoTracker-mitochondrion-selective probes (Invitrogen, USA). Live cell fluorescence images were captured with laser scanning confocal microscope A1 (Nikon, Japan). The nucleus of live cells was stained with Hoechst 33342 (Beyotime, China). Mitochondria from each group were randomly selected and the mitochondrial length was quantified by Image J, the statistical methods were referred from several convincing reports [2-5].

Analysis of mitochondrial bioenergetics and cellular glycolysis

O_2 consumption rate (OCR) (mitochondrial stress test) and extracellular acidification rate (ECAR) (glycolysis stress test) were determined by the Seahorse XF 24-3 analyzer (Agilent, USA). Human PASMCs were plated onto cell culture microplates on the day prior to the experiments. Human PASMCs were seeded in 24-well plates (2000 cells/well) in DMEM with 10% FBS treated with vehicle or CBD (10 μM), with or without $CoCl_2$ (200 μM) for 12 h. The cells were incubated at 37 °C in a CO_2-free XF prep station 60 min before the Seahorse assay to allow the cells to equilibrate with the assay medium. For the mitochondria stress, on the day of the experiment, cells were incubated in XF assay medium (Agilent, USA), supplemented with 25 mM glucose, 1 mM pyruvate for 1 h before the measurement. After the recording of the basal rates of OCR, final concentrations of 1.5 μM oligomycin, 1 μM FCCP and 0.5–0.5 μM rotenone and antimycin A were added (XF Cell Mito Stress Test Kit, Agilent, USA) through the instrument's injection ports. For the glycolysis stress test, after plating and treating human PASMCs properly as described above, 10 mM glucose, 1 μM oligomycin, and 50 mM 2-deoxy-D-glucose (2-DG; glycolysis inhibitor) (XF Cell Glycolysis Stress Test Kit, Agilent, USA) were sequentially injected. Values were normalized by cell staining and counting.

Isolation and quantitation of RNA

Total RNAs were isolated with the TRIzol reagent (Invitrogen, USA). Quantitative real-time RT-PCR (qRT-PCR) was performed on a Light Cycler PCR platform (Roche, USA) in accordance to the manufacturer’s instructions. The genes primer pairs were listed in Table S1.

Western Blot

The protein extraction was separated by 12% sodium dodecyl sulfate-polyacrylamide gel electrophoresis and transferred onto the polyvinylidene difluoride membrane (Millipore, USA), the blots were blocked with 2% non-fat milk at room temperature and incubated with antibody for β-actin (Santa Cruz, USA) or PFKFB3 (Abcam, USA), DRP1 (Santa Cruz, USA), MFN2 (Proteintech, USA), FIS1 (Bioss Antibodies, China) at 4 °C overnight. The blots were incubated with secondary antibody Goat Anti-Rabbit IgG (H+L) HRP (ZSGB-BIO, China) for 1 h at room temperature. The final exposure was detected with an Enhanced Chemiluminescence Detection kit

(Invitrogen, USA) and the density of the bands was analyzed by Image J (USA) software.

References

1. Lee KJ, Czech L, Waypa GB, Farrow KN. Isolation of pulmonary artery smooth muscle cells from neonatal mice. J Vis Exp. 2013: e50889-e.

2. Youle RJ, van der Bliek AM. Mitochondrial fission, fusion, and stress. Science (New York, NY). 2012; 337: 1062-5.

3. Wai T, Langer T. Mitochondrial Dynamics and Metabolic Regulation. Trends in endocrinology and metabolism: TEM. 2016; 27: 105-17.

4. Ryan J, Dasgupta A, Huston J, Chen K-H, Archer SL. Mitochondrial dynamics in pulmonary arterial hypertension. Journal of molecular medicine (Berlin, Germany). 2015; 93: 229-42.

5. Marsboom G, Toth PT, Ryan JJ, Hong Z, Wu X, Fang YH, et al. Dynamin-related protein 1-mediated mitochondrial mitotic fission permits hyperproliferation of vascular smooth muscle cells and offers a novel therapeutic target in pulmonary hypertension. Circulation research. 2012; 110: 1484-97.

Supplemental Table

Table S1. A summary of the qPCR primer sequence

Gene	Source	Forward Primer	Reverse Primer
Actb	mouse	GGCTGTATTCCCCTCCATCG	CCAGTTGGTAACAATGCCATGT
Il6	mouse	TAGTCCTTCCTACCCCAATTTCC	TTGGTCCTTAGCCACTCCTTC
Tnfα	mouse	CCCTCACACTCAGATCATCTTCT	GCTACGACGTGGGCTACAG
Ccl2	mouse	TTAAAAACCTGGATCGGAACCAA	GCATTAGCTTCAGATTTACGGGT
Cxcl10	mouse	CCAAGTGCTGCCGTCATTTTC	GGCTCGCAGGGATGATTTCAA
Hmox1	mouse	AAGCCGAGAATGCTGAGTTCA	GCCGTGTAGATATGGTACAAGGA
Sod1	mouse	AACCAGTTGTGTTGTCAGGAC	CCACCATGTTTCTTAGAGTGAGG
Nfe2l2	mouse	TAGATGACCATGAGTCGCTTGC	GCCAAACTTGCTCCATGTCC
Nqo1	mouse	AGGATGGGAGGTACTCGAATC	AGGCGTCCTTCCTTATATGCTA
Mfn1	mouse	CCTACTGCTCCTTCTAACCCA	AGGGACGCCAATCCTGTGA
Mfn2	mouse	TGACCTGAATTGTGACAAGCTG	AGACTGACTGCCGTATCTGGT
Drp1	mouse	CAGGAATTGTTACGGTTCCCTAA	CCTGAATTAACTTGTCCCGTGA
Keap1	mouse	TGCCCCTGTGGTCAAAGTG	GGTTCGGTTACCGTCCTGC
Pfkfb3	mouse	CCCAGAGCCGGGTACAGAA	GGGGAGTTGGTCAGCTTCG
18s	human	CTTTGGTCGCTCGCTCCTC	CTGACCGGGTTCCTTTTGAT
PFKFB3	human	ATTGCGGTTTTCGATGCCAC	GCCACAACTGTAGGGTCGT
HMOX-1	human	AAGACTGCGTTCCTGCTCAAC	AAAGCCCTACAGCAACTGTCG
SOD1	human	GGTGGGCCAAAGGATGAAGAG	CCACAAGCCAAACGACTTCC
NFE2L2	human	TCAGCGACGGAAAGAGTATGA	CCACTGGTTTCTGACTGGATGT
NQO1	human	GAAGAGCACTGATCGTACTGGC	GGATACTGAAAGTTCGCAGGG
MFN1	human	GAGGTGCTATCTCGGAGACAC	GCCAATCCCACTAGGGAGAAC
MFN2	human	CTCTCGATGCAACTCTATCGTC	TCCTGTACGTGTCTTCAAGGAA
DRP1	human	CTGCCTCAAATCGTCGTAGTG	GAGGTCTCCGGGTGACAATTC
FIS1	human	GATGACATCCGTAAAGGCATCG	AGAAGACGTAATCCCGCTGTT
OPA1	human	CGACCCCAATTAAGGACATCC	GCGAGGCTGGTAGCCATATTT
PDK1	human	CTGTGATACGGATCAGAAACC	TCCACCAAACAATAAAGAGTGC

		G	T
KEAP1	human	CTGGAGGATCATACCAAGCAG<br>G	GGATACCCTCAATGGACACCAC
MIEF1	human	CACGGCCATTGACTTTGTGC	TCGTACATCCGCTTAACTGCC

Supplemental Figures

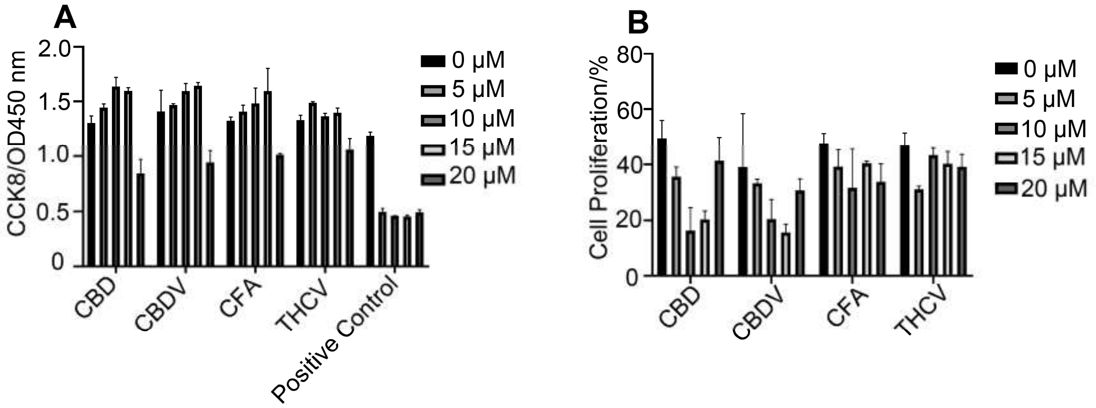

Figure S1 Screening of the cytotoxicity and anti-proliferative effect among several cannabinoid compounds

A, Cell viability assessed by CCK8 assay, 24 h after the mice PASMCs treated with the CBD, CBDV, CFA, THCV at concentration of 5 μM, 10 μM, 15 μM, 20 μM and positive control (0% or 10% ethanol) (n = 6 per group). **B**, Quantitative assessment of BrdU antibody to calculate the ratio of mice PAH-PASMCs proliferation, treated with CBD, CBDV, CFA, THCV at 5 μM, 10 μM, 15 μM, 20 μM concentration and positive control (0% or 10% ethanol) (n = 7 per group).

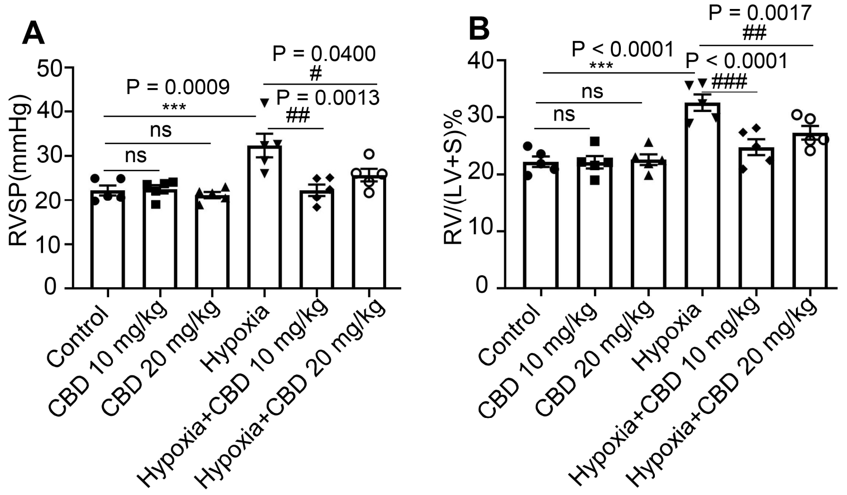

Figure S2 Different concentration of CBD treatment in Sugen-hypoxia PAH preventive mouse model.

A and B, RVSP and RVH of Sugen-hypoxia induced PAH mouse models were assessed, grouped by with or without hypoxia treatment and 10 mg/kg or 20 mg/kg CBD daily intragastric administration, n = 5 per group. The results were analyzed by one-way ANOVA followed by Bonferroni's multiple comparison test, $^{*}P < 0.05$, $^{**}P < 0.01$, $^{***}P < 0.001$ vs. the control group, and $^{\#}P < 0.05$, $^{\#\#}P < 0.01$, $^{\#\#\#}P < 0.001$ vs. the hypoxia treatment group.

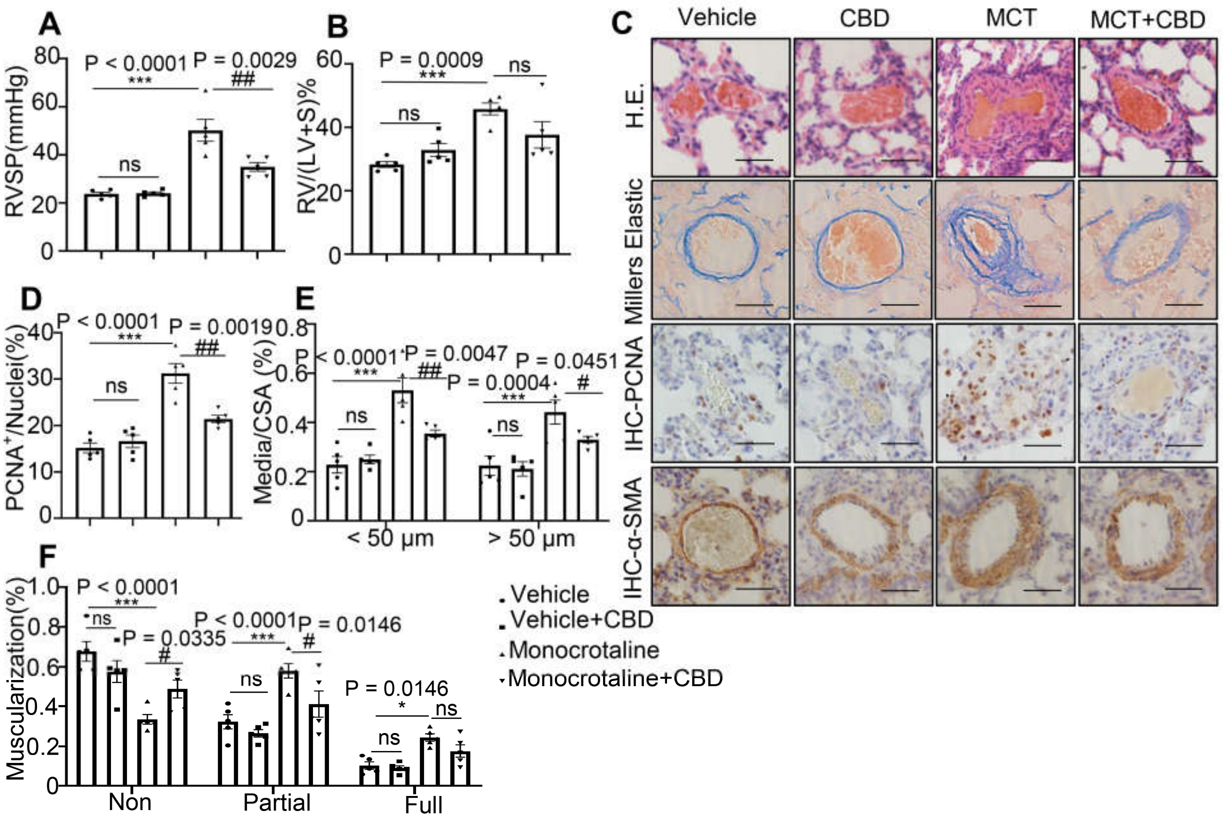

Figure S3 CBD treatment of MCT-induced PAH preventive rat model.

A and **B**, Assessments of RVSP and RVH. **C-F**, Representative images of pulmonary arteries stained with H&E, and representative images of vascular remodeling in the distal arterioles stained with elastin or immunostained for PCNA and α-SMA. Pulmonary vascular remodeling rate in the MCT PAH rats, including the quantification of the relative number of $PCNA^+$/nuclei (**D**), the degree of medial wall thickness as a ratio of total vessel size (Media/CSA) (**E**), and the proportion of non-, partially-, or fully muscularized pulmonary arterioles (25 to 75 μm in diameter) from PAH model rats (**F**) (n = 10 per group). Scale bar = 20 μm. The results were analyzed by one-way ANOVA followed by Bonferroni's multiple comparison test, $^{*}P < 0.05$, $^{**}P < 0.01$, $^{***}P < 0.001$ vs. the control group, and $^{\#}P < 0.05$, $^{\#\#}P < 0.01$, $^{\#\#\#}P < 0.001$ vs. the MCT treatment group.

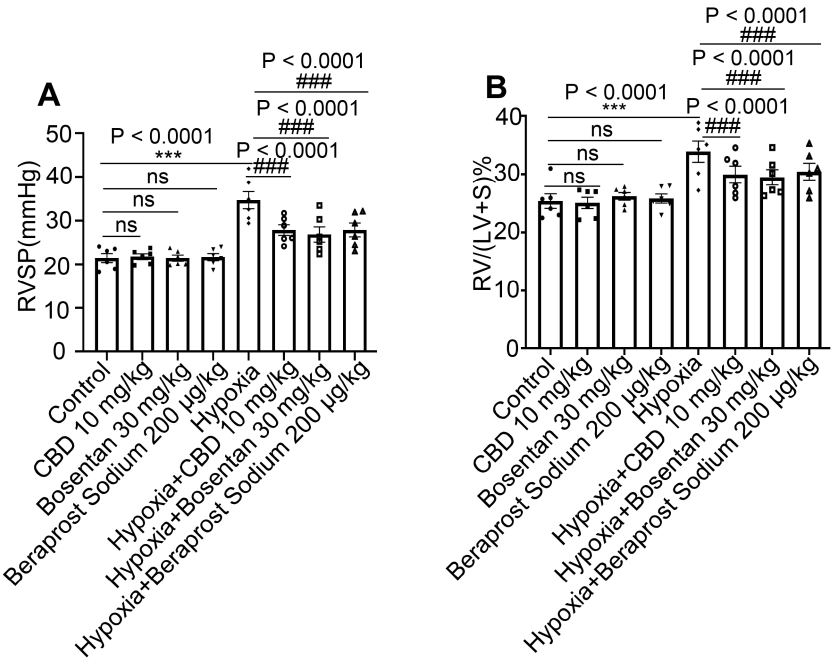

Figure S4 Comparison of the efficacy of CBD, bosentan and beraprost sodium in the hypoxia-induced preventive PAH mice.

A and B, RVSP and RVH of Sugen-hypoxia-induced PAH mouse models were assessed, grouped by with or without hypoxia treatment and 10 mg/kg CBD, bosentan (30 mg/kg) daily intragastric administration and beraprost sodium (200 μg/kg, intravenous (i.v.) injection) once per week for 3 weeks, n = 5 per group. The results were analyzed by one-way ANOVA followed by Bonferroni's multiple comparison test, $^{*}P < 0.05$, $^{**}P < 0.01$, $^{***}P < 0.001$ vs. the control group, and $^{\#}P < 0.05$, $^{\#\#}P < 0.01$, $^{\#\#\#}P < 0.001$ vs. the hypoxia treatment group.

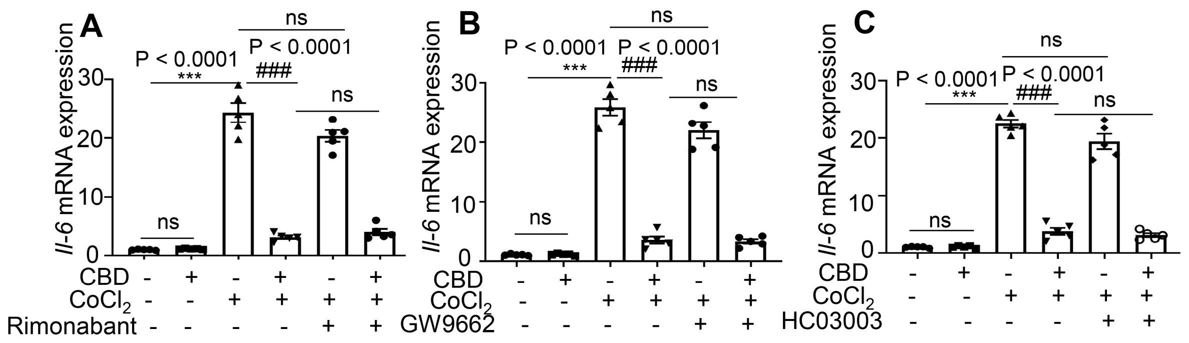

Figure S5 CBD reduced the expression of *Il6* without the participation of several cannabinoids' receptors in mice PASMCs.

A-C, mRNA level of *Il6* treated by $CoCl_2$, and the effect of antagonist or channel blocker of cannabinoid receptors (Rimonabant, GW9662 and HC030031) in mice PASMCs, the concentration of them were 10 μM, which were equal to the concertation of CBD, the antagonists were pre-treated for 30 min before CBD added, n = 6 per group. The results were analyzed by one-way ANOVA followed by Bonferroni's multiple comparison test, $^{*}P < 0.05$, $^{**}P < 0.01$, $^{***}P < 0.001$ vs. the control group, and $^{\#}P < 0.05$, $^{\#\#}P < 0.01$, $^{\#\#\#}P < 0.001$ vs. the $CoCl_2$ treatment group.

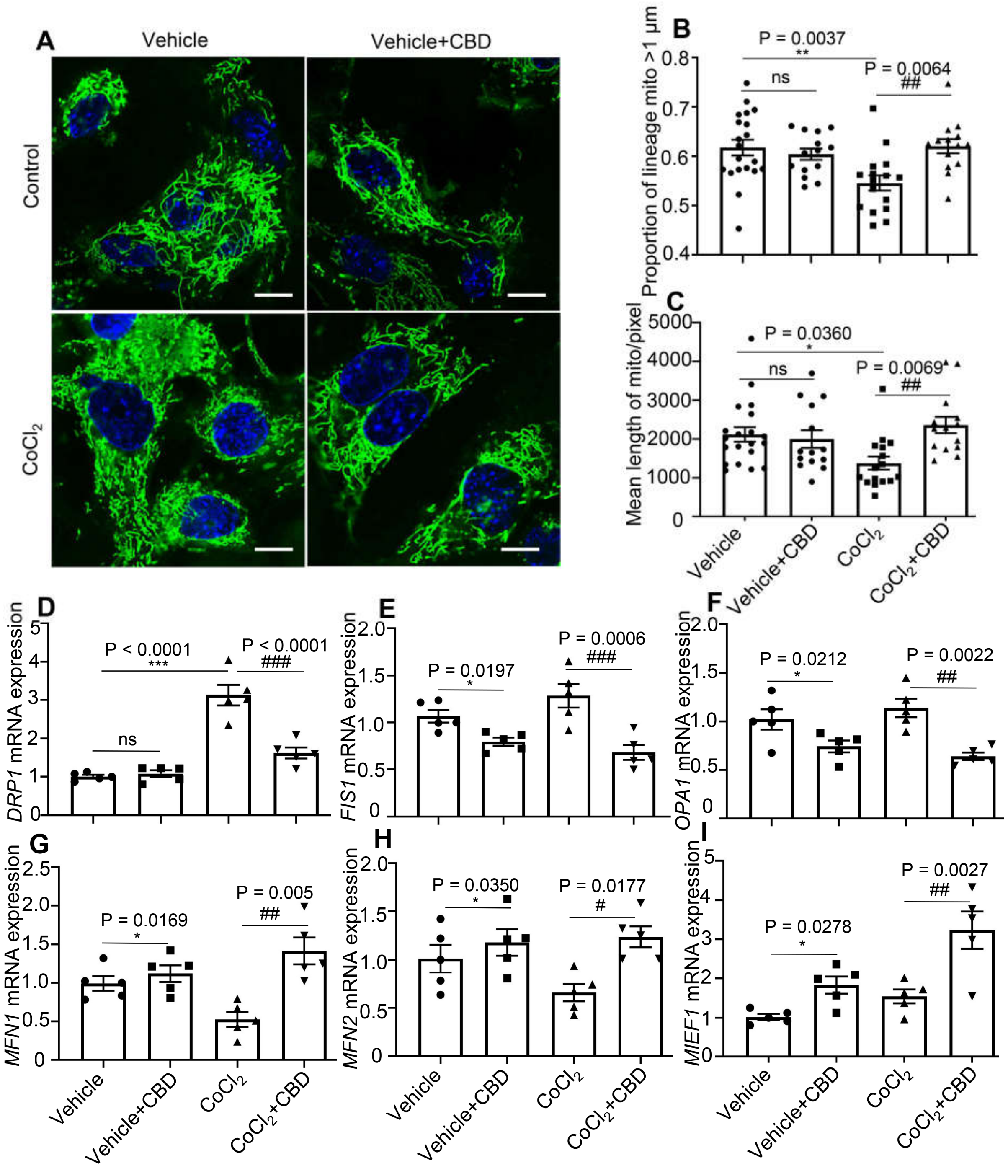

Figure S6 CBD treatment on mitochondrial networks in human PAH-PASMCs.

A, Representative images of control human PASMCs and $CoCl_2$ treated human PASMCs labeled with MitoTracker after the treatment with CBD (10 µM) or the control vehicle for 2 h. Nuclei were counterstained using Hoechst 33342, scale bar = 10 µm. **B** and **C**, Quantification of mitochondrial proportion of lineage that longer than 1 µm (40 pixels, about 1µm) and mean length of mitochondria/pixels in control human PASMCs and $CoCl_2$ treated human PASMCs labeled with MitoTracker after the treatment with CBD or the control vehicle for 2 h, n = 20 per group. **D-I**, Real-time qPCR analyses for expression levels of *DRP1, FIS1, OPA1, MFN1, MFN2, MIEF1* in $CoCl_2$ treated human PASMCs after the treatment with CBD (10 µM) or vehicle for 12 h. n = 6 per

group. The results were analyzed by one-way ANOVA followed by Bonferroni's multiple comparison test, *$P < 0.05$, **$P < 0.01$, ***$P < 0.001$ vs. the control group, and $^{\#}P < 0.05$, $^{\#\#}P < 0.01$, $^{\#\#\#}P < 0.001$ vs. the $CoCl_2$ treatment group.

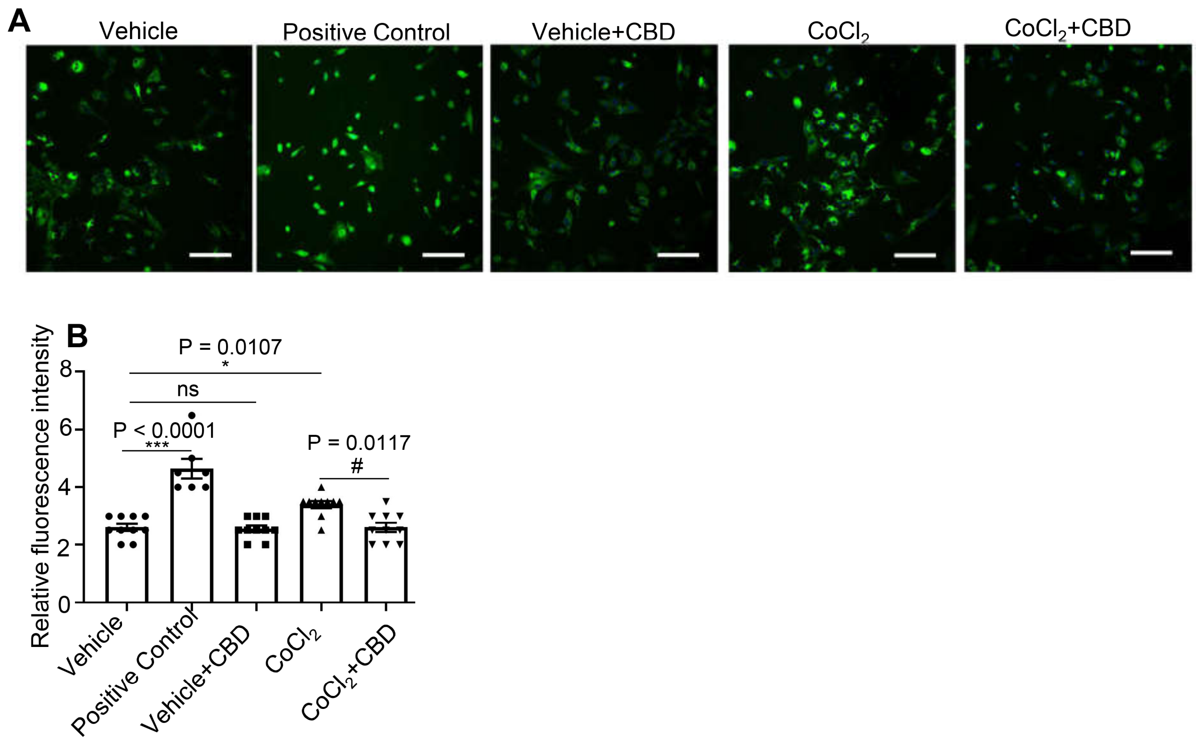

Figure S7 Normalization of CBD on cellular ROS of human PASMCs.

A, Quantification of ROS with a fluorescence 96-plate by a fluorescence microplate reader and DCFH-DA fluorescence intensity in human PASMCs with or without $CoCl_2$ and/or CBD treatment or rosup (provided in the kit as a positive control) for 2 h, n = 8 per group. **B**, Representative images of ROS fluorescence assessed by laser scanning microscope in human PASMCs with or without $CoCl_2$ and/or CBD treatment. Nuclei was counterstained using DAPI, n≥7 per group. Scale bar = 100 μm. The results were analyzed by one-way ANOVA followed by Bonferroni's multiple comparison test, $*P < 0.05$, $**P < 0.01$, $***P < 0.001$ vs. the control group, and $^{\#}P < 0.05$, $^{\#\#}P < 0.01$, $^{\#\#\#}P < 0.001$ vs. $CoCl_2$ treatment group.

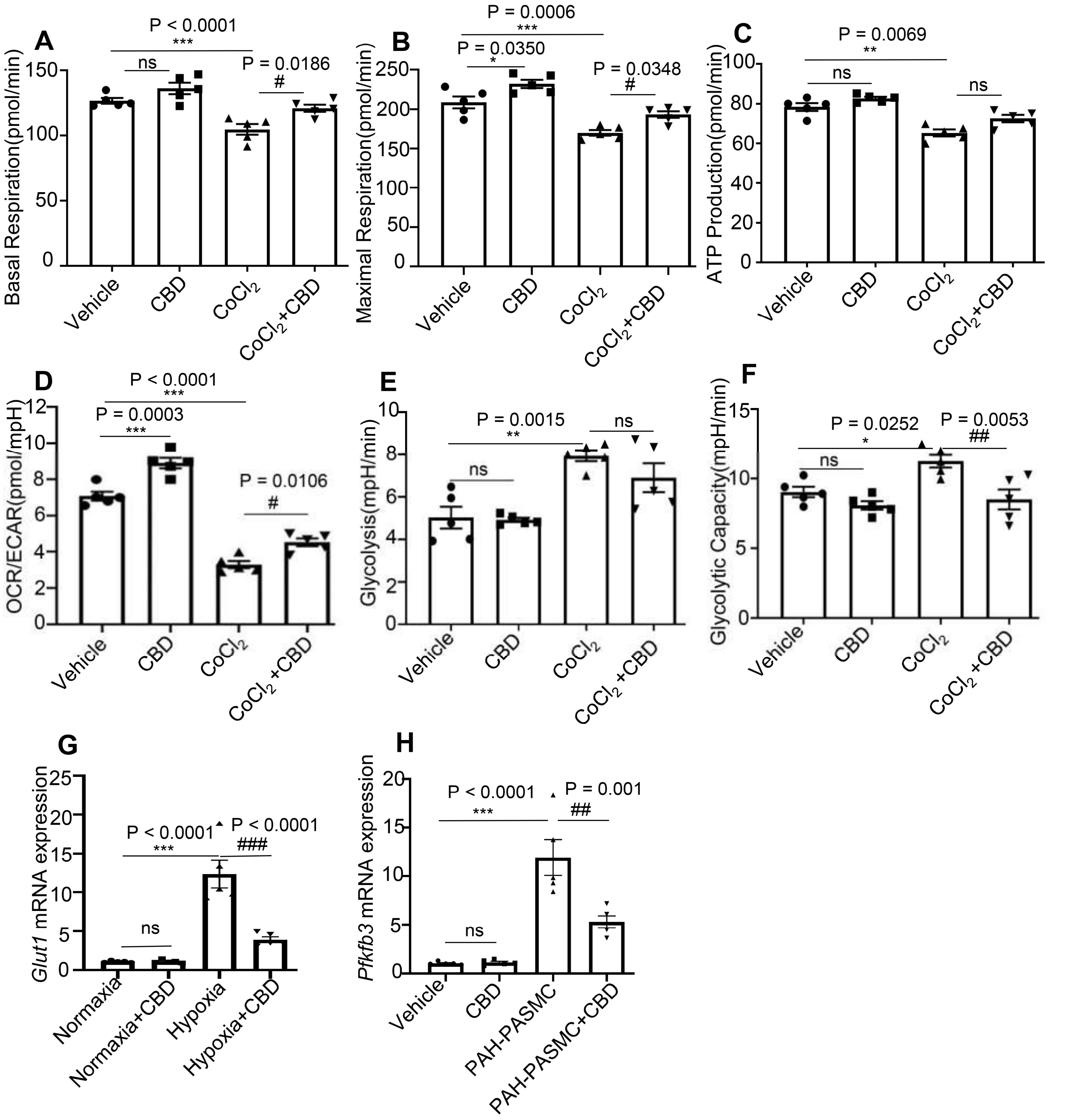

Figure S8 CBD can reverse hypoxia-induced abnormal glycolysis in both human PASMCs and PAH mice.

A-D, Quantification of the OCR and ECAR in human PASMCs after treatment with CBD or vehicle for 12 h, n = 5 per group. Data assessed by mitochondria stress test, including cellular basal respiration, maximal respiration, ATP production and OCR/ECAR. **E** and **F**, Summarized data from glycolytic stress test showing the basal glycolytic rate and the maximal glycolytic capacity. **G**, mRNA level of glycolysis marker *Glut1* in the lungs from preventive PAH mice, n = 5 per group. **H**, mRNA level of *Pfkfb3* in mice PAH-PASMCs with or without CBD treatment, n = 5 per group. The results were analyzed by one-way ANOVA followed by Bonferroni's multiple comparison test, $*P <$

0.05, ** $P < 0.01$, ***$P < 0.001$ vs. the control group, and $^{\#}P < 0.05$, $^{\#\#}P < 0.01$, $^{\#\#\#}P < 0.001$ vs. the PAH-PASMCs or the hypoxia-induced mice.
